# Insights on the virulence of swine respiratory tract mycoplasmas through genome-scale metabolic modeling

**DOI:** 10.1186/s12864-016-2644-z

**Published:** 2016-05-13

**Authors:** Mariana G. Ferrarini, Franciele M. Siqueira, Scheila G. Mucha, Tony L. Palama, Élodie Jobard, Bénédicte Elena-Herrmann, Ana T. R. Vasconcelos, Florence Tardy, Irene S. Schrank, Arnaldo Zaha, Marie-France Sagot

**Affiliations:** ERABLE, Inria, 43, Bd du 11 Novembre 1918, Villeurbanne, France; CBiot, UFRGS, Av Bento Gon’calves, Porto Alegre, 9500 Brazil; Université de Lyon, Institut des Sciences Analytiques (CNRS, ENS Lyon, Université Lyon 1), 5, Rue de la Doua, Villeurbanne, France; Laboratório Nacional de Computaćão Científica, Av. Getúlio Vargas, 333, Petrópolis, Brazil; Anses, Laboratoire de Lyon, UMR Mycoplasmoses des Ruminants, 31, Av Tony Garnier, Lyon, France; Laboratoire de Biométrie et Biologie Évolutive, Université de Lyon, 43, Bd du 11 Novembre 1918, Villeurbanne, France; Université de Lyon, Centre Léon Bérard, Département d’oncologie médicale, 28, rue Laënnec, Lyon, France; Université de Lyon, VetAgro Sup, UMR Mycoplasmoses des Ruminants, 1 Avenue Bourgelat, Marcy L’Étoile, France; Current address: LISBP – INSA Toulouse, Toulouse, France

**Keywords:** *Mycoplasma*, *Mollicutes*, Metabolic network, Metabolism, Whole-genome metabolic reconstruction, Hydrogen peroxide

## Abstract

**Background:**

The respiratory tract of swine is colonized by several bacteria among which are three *Mycoplasma* species: *Mycoplasma flocculare*, *Mycoplasma hyopneumoniae* and *Mycoplasma hyorhinis*. While colonization by *M. flocculare* is virtually asymptomatic, *M. hyopneumoniae* is the causative agent of enzootic pneumonia and *M. hyorhinis* is present in cases of pneumonia, polyserositis and arthritis. The genomic resemblance among these three *Mycoplasma* species combined with their different levels of pathogenicity is an indication that they have unknown mechanisms of virulence and differential expression, as for most mycoplasmas.

**Methods:**

In this work, we performed whole-genome metabolic network reconstructions for these three mycoplasmas. Cultivation tests and metabolomic experiments through nuclear magnetic resonance spectroscopy (NMR) were also performed to acquire experimental data and further refine the models reconstructed *in silico*.

**Results:**

Even though the refined models have similar metabolic capabilities, interesting differences include a wider range of carbohydrate uptake in *M. hyorhinis*, which in turn may also explain why this species is a widely contaminant in cell cultures. In addition, the myo-inositol catabolism is exclusive to *M. hyopneumoniae* and may be an important trait for virulence. However, the most important difference seems to be related to glycerol conversion to dihydroxyacetone-phosphate, which produces toxic hydrogen peroxide. This activity, missing only in *M. flocculare*, may be directly involved in cytotoxicity, as already described for two lung pathogenic mycoplasmas, namely *Mycoplasma pneumoniae* in human and *Mycoplasma mycoides* subsp. *mycoides* in ruminants. Metabolomic data suggest that even though these mycoplasmas are extremely similar in terms of genome and metabolism, distinct products and reaction rates may be the result of differential expression throughout the species.

**Conclusions:**

We were able to infer from the reconstructed networks that the lack of pathogenicity of *M. flocculare* if compared to the highly pathogenic *M. hyopneumoniae* may be related to its incapacity to produce cytotoxic hydrogen peroxide. Moreover, the ability of *M. hyorhinis* to grow in diverse sites and even in different hosts may be a reflection of its enhanced and wider carbohydrate uptake. Altogether, the metabolic differences highlighted *in silico* and in vitro provide important insights to the different levels of pathogenicity observed in each of the studied species.

**Electronic supplementary material:**

The online version of this article (doi:10.1186/s12864-016-2644-z) contains supplementary material, which is available to authorized users.

## Background

The respiratory tract of swine is colonized by several bacteria, among which are three common *Mycoplasma* species: *Mycoplasma flocculare*, *Mycoplasma hyopneumoniae*, and *Mycoplasma hyorhinis* [1–3]. Even though little information is available concerning the prevalence of bacteria in healthy lungs, these three *Mycoplasma* species have been isolated from the respiratory tract of both healthy and diseased pigs [4–6].

While *M. flocculare* is usually described as a commensal bacterium [7], *M. hyopneumoniae* and *M. hyorhinis* are considered pathogenic. Enzootic pneumonia, caused by *M. hyopneumoniae*, is widespread in pig populations and is considered a major cause of economic loss in the pig industry [8]; *M. hyorhinis* is frequently present in cases of polyserositis and arthritis and *M. flocculare* has high prevalence in swine herds worldwide, but up to date, no disease has been *per se* associated with this species [7]. In addition to these mycoplasmas, *Mycoplasma hyosynoviae*, the primary agent of non-purulent arthritis, can occasionally colonize in the lower respiratory tract when pneumonic lesions pre-exist [9].

Besides mycoplasmal pneumonia, the Porcine Respiratory Disease Complex (PRDC) has emerged as an economically signigicant respiratory disorder characterized by the slow growth, fever, cough, loss of appetite, lethargy and dyspnea in pigs [10, 11]. Even though many species are related to PRDC, it is essential to note that enzootic pneumonia caused by *M. hyopneumoniae* is by far the most costly disease in pig industry, and this bacteria is usually seen as an essential component to the successful establishment of a pathogenic community in the host [12]. Also, *M. hyopneumoniae* infections take longer to cause lesions and to be successfully eliminated than infections from other pathogens [10].

While mycoplasmal diseases in swine have been extensively studied, their causative agents have not been explored from a mathematical and computational point of view, mostly because their genome sequences were not available until recently [13–21]. Although recent studies have placed *M. hyopneumoniae*, *M. flocculare* and *M. hyorhinis* in close proximity within the hyopneumoniae clade by phylogenomic analysis [18], which corroborates with their high 16S rRNA sequence similarity [22], it is not yet clear what causes the specific pathogenicity or lack thereof in each of them. This elevated genomic resemblance combined with their different levels of pathogenicity is an indication that these species, as for most mycoplasmas, have unknown mechanisms of virulence and differential expression.

Pathogenic determinants such as adhesion to the host cell and evasion from the immune response have already been well-described in the literature for both *M. hyopneumoniae* and *M. hyorhinis* [23–27]. The presence of a capsule in *M. hyopneumoniae* has been reported to be important for the interaction with the host cells in one single study [28]. Several studies also show that immunosuppressed animals experimentally infected with *Mycoplasma* species develop less severe microscopic lesions of pneumonia if compared to normal animals [29–31]. This means that it is possible in some cases that a strong response from the host immune system might be the primary cause of pathogenesis. However, up to date, it is not possible to draw any further conclusions due to lack of experimental data.

Even if these topics are of utter importance for understanding swine respiratory tract mycoplasmal diseases, what has yet to be better understood is the direct participation of metabolism in the development of them. For instance, although adhesion factors are related to pathogenicity, *M. hyopneumoniae* and *M. flocculare* harbor similar sets of adhesion proteins [32], and have been shown to adhere to cilia in a similar way [33]. Thus, the ability of *M. hyopneumoniae* to cause disease if compared to *M. flocculare* might not be directly related to adhesion.

Furthermore, the genome sizes of *Mycoplasma* spp. range from 580 kb (*Mycoplasma genitalium*) to more than 1,358 kb (*Mycoplasma penetrans*), representing an important example of genome reduction (and minimal metabolism) during the evolutionary process. It is possible that in an initial symbiotic phase, the host provided a broad range of metabolites for these bacteria. This, together with the ability of the bacteria to uptake such compounds, made several activities dispensable for the bacterial life. Over the course of evolution, these bacteria would have lost some of the genes that became unnecessary for life in an environment conditioned by another genome [34].

In this way, we aimed at studying the reduced metabolism through genome-scale metabolic model reconstructions of *M. hyopneumoniae*, *M. hyorhinis* and *M. flocculare* to better understand their different life-styles. Based on the reconstructed networks, we propose that one of the mechanisms that may explain why *M. hyorhinis* and *M. hyopneumoniae* are pathogenic while *M. flocculare* is not, is their ability to use glycerol as a carbon source, thus enabling the production of the highly toxic hydrogen peroxide. This trait may be directly involved in cytotoxicity, as already described for two lung pathogenic mycoplasmas, namely *Mycoplasma pneumoniae* in human [35] and *Mycoplasma mycoides* subsp. *mycoides* in ruminants [36].

Additionally, growth rates and fitness have been correlated in the past to virulence in several other organisms [37–39] and may be a key factor for the difference in pathogenicity among these three mycoplasmas. Indeed, the *in silico* models show that *M. hyorhinis* and *M. hyopneumoniae* have extra sets of enzymes that may help them overgrow the non-pathogenic species *M. flocculare*. *M. hyorhinis* seems to have a wider carbohydrate uptake range, and probably for this reason is seen as a well-known contaminant of cell cultures [40]. *M. hyopneumoniae*, on the other hand, might use myo-inositol as a carbon source producing acetyl-coenzyme-A as a byproduct. This feature is of particular interest for the production of cofactor Coenzyme-A in this species, since most enzymes from the biosynthetic pathway are missing in all species.

Nevertheless, there is little metabolic experimental data available for the three species, which makes the reconstruction of a reliable metabolic model an extremely time-consuming work. Together with the fact that these mycoplasmas are generally grown in complex media, with high serum concentrations, we needed additional experimental data in order to compare the *in silico* reconstructed metabolic networks with the in vivo metabolic characteristics of the three species. For this reason, we also performed nuclear magnetic resonance spectroscopy (NMR) analyses to detect metabolites consumed and produced in both complex and defined media. These experiments corroborated with the reconstructed models and suggested two new features in particular: (i) the uptake of myo-inositol in *M. hyopneumoniae* might be related to a higher acetate production, and (ii) *M. hyorhinis* showed a surprisingly reduced ability to convert pyruvate to acetate in the growth conditions used in this study. All these *in silico* and in vivo metabolic differences might influence the different levels of pathogenicity in each of the species studied here.

We present in this work the metabolic networks reconstructed from the annotated genomes of the three species and the comparison done with growth rates and metabolomic experiments performed in vitro in order to better understand the basis of the pathologies caused by these bacteria, which might help prevent their development in the future.

## Methods

### Modeling methods

#### Network reconstruction and refinement

We reconstructed the metabolic networks for all available strains isolated from swine so far of *M. hyopneumoniae* (pathogenic strains 232, 7422, 7448, 168 and non-pathogenic strains J and 168L), *M. hyorhinis* (HUB-1, GDL-1, SK-76 and ATCC 17981 strain BTS7) and *M. flocculare* (ATCC 27716 and ATCC 27399). The semi-automated reconstructions were generated by the Pathologic tool from the Pathway Tools software [41] using the complete genomes available online. From now on, the species strains will be abbreviated according to Table 1.

**Table 1:** Selected species and strains, abbreviation, NCBI accession numbers and experimental availability of strains

Species	Strain	Accession number	Abbreviation	Pathogenicity level	Experimental availability ⋆^*a*^	Reference
*M. hyopneumoniae*	168	NC_017509	MHP_168	Pathogenic	NA	[16]
	168L	CP003131	MHP_168L	Attenuated	NA	[19]
	232	AE017332	MHP_232	Pathogenic	NA	[13]
	7422	NC_021831	MHP_7422	Pathogenic	Brazil	[18]
	7448	AE017244	MHP_7448	Pathogenic	Brazil	[14]
	J	AE017243	MHP_J	Attenuated	Brazil and France	[14]
	All Strains	NA	MHP	NA	NA	NA
*M. hyorhinis*	HUB-1	NC_014448	MHR_HUB1	Pathogenic	NA	[15]
	GDL-1	NC_016829	MHR_GDL1	NA ⋆^*b*^	NA	[17]
	SK-76	NC_019552	MHR_SK76	Pathogenic	NA	[20]
	ATCC 17981	ARTL0100000	MHR_17981	Attenuated	Brazil and France	NA ⋆^*c*^
	All Strains	NA	MHR	NA	NA	NA
*M. flocculare*	ATCC 27399	CP007585	MFL_27399	Commensal	France	[21]
	ATCC 27716	AFCG01000000	MFL_27716	Commensal	Brazil	[18]
	All Strains	NA	MFL	NA	NA	NA

Notes:

NA: Not available

⋆^*a*^: Strains available only in Brazil are protected strains which are property of the government and cannot be used outside authorised laboratories. Strains available in France were purchased from the ATCC repository; these strains were also available for testing in the laboratory in Brazil

⋆^*b*^: *M. hyorhinis* strain GDL-1 was retrieved from a contaminated cell line and, to our knowledge it has never been reported neither as pathogenic nor as an attenuated strain

⋆^*c*^: *M. hyorhinis* strain ATCC 17981 genome is available online but not published in any paper up to now. (source: http://genomeportal.jgi.doe.gov/Mychy1/Mychy1.info.html)

Pathway Tools automatically associated genes with reactions, based on the annotation names, Gene Ontology (GO) terms and enzyme code (EC) numbers contained in the GenBank files. The software assembled reactions into pathways by comparing them with the reference database, MetaCyc. The software automatically added missing reactions from the reference database to the model, creating many orphan reactions (reactions that do not have an enzyme associated). In a gapfilling step, we used the Pathway Hole Filler tool, included in the Pathway Tools software, to identify possible candidate genes associated with these orphan reactions. Orphan reactions in the metabolic network were allowed only to allow known functionalities of the organisms or biomass production in the reconstructed models. We incorporated the minimal number of orphan reactions into the models to avoid adding a behavior that was never described.

In our work, the refinement of the networks was made in a subsystems approach [42] simultaneously on all organisms. As a result of different genomic annotations, slightly different reactions arose throughout the automatic models. These reactions were checked for consistency, and after validation of only one of them, the duplicates were deleted. Gene-Protein-Reaction (GPR) associations were systematically validated or included based on experimental data, information provided by the Pfam functional domain database [43], synteny analysis (gene context) and reciprocal sequence homology (BLASTp) searches, using at first high E-value cutoffs of at least 10^−10^ [44]. Whenever no hits were achieved with these parameters, more relaxed parameters were assumed (E-value cutoffs of at least 10^−5^). Homolog proteins with equivalent functional domains were assigned as isozymes to a particular reaction, while proteins with distinct functional domains were assigned as subunits of a multi-protein complex.

Non-metabolic reactions such as DNA polymerization, protein synthesis and RNA synthesis were explicitly deleted from the network but implicitly included in the biomass assembly. Generic reactions were either specified or excluded from the models. The resulting models were further refined in accordance with a detailed protocol from Palsson and Thiele [45]. Reactions were computationally balanced for mass and charge, while cofactor usage was determined based on literature data for closely related species [46–48]. Reaction directionality was thermodynamically checked and validated. Reaction directionality was determined based on the component contribution method [49, 50], which extends the group contribution method [51] and achieves a significant improvement in the accuracy of the estimations of standard Gibbs energies. An online search and calculation interface called eQuilibrator along with metabolite and reaction thermodynamic databases are available at [52, 53]. Heuristic rules were used to improve the directionality assignment.

We analyzed the network topology to identify compounds that were only produced or consumed in the network, the so-called dead-end metabolites (DEM). Whenever a DEM was found, we either (i) added or validated an orphan reaction to reconnect it to the rest of the network, or (ii) removed the reaction from the model when both substrate(s) and product(s) were disconnected from the network and/or did not affect the overall metabolism. Transporters were predicted at first from genome annotation using the Transport Identification Parser, from Pathway Tools [54]. Transporters were predicted mainly based on sequence similarity to other known transporters, since experimental data for the three mycoplasmas were not available. Specific transport reactions and exchange reactions required for production of biomass components were manually added to the final version of the networks.

#### Biomass composition and biomass equation

In order to simulate growth, we had first to estimate the average cell composition of these mycoplasmas (biomass composition). To this end, we used a general macromolecule mycoplasma cell composition from Razin and collaborators [55]. Depending on the species, the authors showed that the solid residue contained 54–62 % of proteins, 12–20 % of lipids, 3–8 % of carbohydrates, 8–17 % of RNA, and 4–7 % of DNA. The membranes comprised around 35 % of dry weight of the organisms and contained 47–60 % of proteins, 35–37 % lipids, 4–7 % carbohydrates and small amounts of DNA and RNA. Since no information on metal ions and cofactors was available, we included them quantitatively based on the metabolic networks reconstructed for related mycoplasmas [46, 47]. Membrane and lipid components were added based on literature composition of the selected species. It is important to note that the only difference in composition between the species reported so far was related to the presence of glycolipids in *M. hyopneumoniae* and *M. flocculare*, which were reported to be absent in *M. hyorhinis* [56–58]. Based on the previous information, we assumed the following fractions of macromolecules: 55 % proteins, 15 % lipids, 6.88 % carbohydrates, 12 % RNA, 6 % DNA, and 5.12 % of ions and cofactors. To create a biomass elemental formula, we took into account the percentage contribution of each of the components to the overall cell. The biomass reaction represented the drain of these components into biomass production. It implicitly assembled DNA replication, RNA transcription, and protein synthesis into one single reaction. Amino acids were indirectly included in the biomass reaction: charged tRNAs were accounted as substrates and uncharged tRNAs as products. Growth and non-growth associated maintenance (GAM and NGAM) were estimated based on the literature and were manually added to the models [59, 60]. The details for the complete assembly of the biomass reaction can be found in Additional file 2.

#### Model validation and FBA analysis

The metabolic networks were exported as a mathematical Systems Biology Markup Language (SBML) models [61]. They were uploaded to the COBRA-toolbox v2.0 Matlab extension [62] for Flux Balance Analysis (FBA) testing. Minimum-maximum flux constraints were imposed based on literature information [46]. Growth simulations were achieved using biomass production as the FBA objective function.

### Experimental methods

#### Swine mycoplasmas cultivation

Complex media comprised (i) Friis media [63] (available in Brazil), and (ii) a commercial mycoplasma broth (provided by Indicia Biotechnology, available in France). Defined media cultivation tests were performed in France and comprised (i) a medium described for *M. pneumoniae* strain 129 by Yus and collaborators (2009), and (ii) commercial media CMRL with no glutathione (Invitrogen). Since we had no information on metabolism of swine mycoplasmas, we decided to supplement the defined Yus medium with all amino acids (from now on the supplemented version will be referred to as Yus+). We also supplemented the CMRL-1066 medium with other peptone and/or other cofactors (from now on these will be referred to as CMRL+ and CMRL+/Pep). Information on the composition of all defined media is available in Additional file 1: Table S1. Cells were cultivated at 37 °C for different time periods, under gentle agitation (100 *rpm*).

#### Cell concentration estimation and viability by color changing units (CCU) measurement

Cell growth and viability was measured with triplicate time-matched samples of cells and culture media for CCU as described by Stemke and Robertson [64]. Viability of cells is visible by a change in medium color from red to yellow. For cell concentration measurements, the cultures were subjected to a series of 10-fold dilutions in complex media and 1 CCU/*mL* was defined as the highest dilution of cells able to change the medium color [65].

#### Samples for NMR spectroscopy

NMR was performed with complex Friis medium (with strains MHP_7448, MHP_J, MHR_17981 and MFL_27716) and defined Yus+ medium (with strains MHP_J, MHR_17981 and MFL_27716). The medium was collected at the following time intervals: 0 *h*, 8 *h*, 10 *h*, 24 *h*, 32 *h*, and 48 *h* for Friis medium and 0 *h*, 8 *h*, 24 *h*, 32 *h*, 48 *h*, 56 *h* and 72 *h* for Yus+ medium. Cells were separated from growth media through sedimentation at 3360 *g* for NMR analysis. Samples consisted of biological triplicates in complex medium and biological duplicates in defined medium. Since we had slight different time intervals, care was taken by not directly comparing the results from different experiments.

#### NMR analysis

Sample preparation was as follows: 60 *μL* of a mixture containing 1.25 *MKH*_2_*PO*_4_ phosphate buffer (pH=7,4) in *D*_2_*O* with 2 *mMNaN*_3_ and 0.1 % trimethylsilyl propionate (TMSP) was added to 540 *μL* supernatant samples. Both solutions were mixed thoroughly and 550 *μL* were then transferred to 5 *mm* NMR tubes and sorted in 96-tubes racks.

All NMR experiments were carried out on a Bruker 800 *MHz* NMR spectrometer equipped with a 5 *mm* TXI probe and a SampleJet autosampler, enabling high-throughput data acquisition for large collections of samples. The temperature was controlled at 27 °C throughout the experiments, and the samples were kept refrigerated at 4 °C during a waiting time of less than 24 *h* in the autosampler, before the NMR analysis. Standard ^1^H 1D NMR pulse sequence nuclear Overhauser effect spectroscopy (NOESY) with z-gradient and Carr-Purcell-Meiboom-Gill (CPMG) with water presaturation (Bruker pulse program noesygppr1d and cpmgpr1d) were applied on each sample to obtain the corresponding metabolic profiles. A total of 128 transient free induction decays (FID) were collected for each experiment with a spectral width of 20 *ppm*. The relaxation delay was set to 4 *s*. The NOESY mixing time was set to 10 *ms*. The total acquisition time of each sample was 12 *min* 34 *s*. The CPMG spin-echo delay was set to 300 ms, for a total filter of 77 ms, allowing an efficient attenuation of the lipid and protein NMR signals. The 90° pulse length was automatically calibrated for each sample at around 10 *μs*.

Data processing: All FIDs were multiplied by an exponential function corresponding to a 0.3 *Hz* line-broadening factor, prior Fourier transformation. 1H-NMR spectra were manually phased and referenced to the TSP signal (*δ*= - 0.016 *ppm* at pH 7.4) using Topspin 3.1 (Bruker GmbH, Rheinstetten, Germany). Extraction of a data matrix for multivariate statistical analysis from the ^1^H NMR profiles was done using the Statistics toolbox of AMIX (Bruker Biospin). Spectra were integrated from 0.3 to 10 *ppm* at a step of 0.01 *ppm* but excluding the regions of residual water at 4.68-4.88 *ppm*. No normalization of the intensity was performed. The resulting data matrix contains 947 NMR variables.

Multivariate data analysis: Principal component analysis (PCA) and hierarchical clustering analysis (HCA) were performed using SIMCA-P 13 (Umetrics, Umea, Sweden) with scaling based on the Pareto method.

Metabolites identification and quantification: Metabolite identification was achieved by comparing spectra with databases such as HMDB [66]. Identification of the metabolites was further verified with homonuclear and heteronuclear 2D NMR experiments such as ^1^*H*−^1^3C HSQC, ^1^*H*−^1^H TOCSY and J-resolved experiments. Absolute quantification of the metabolites was performed using Chenomx NMR Suite (Chenomx Inc., Edmonton, Canada).

## Results

### Model reconstruction and refinement

Based on the published genomes of 6 strains of *M. hyopneumoniae*, 4 strains of *M. hyorhinis* and 2 strains of *M. flocculare*, we reconstructed 16 genome-scale metabolic models: one model for each strain separately, one for each species and a pan-reconstruction for all three species (Table 1). The semi-automated reconstructions were manually refined according to the description given in the “Methods” section.

The Pan-swine *Mycoplasma* network (representing the merge of all strains, and called pan-network) was initially composed by 829 reactions. Duplicate reactions arose from the fact that Pathway Tools is based only on the annotations contained in the GenBank files. We removed generic and duplicate reactions from the models and replaced them with the specific and validated ones. Non-metabolic reactions were also excluded at this point, along with absent capabilities of these mycoplasmas, such as heme, quinone or cytochrome dependent reactions [67]. The networks were also tested for the presence of DEM and the ability to produce all biomass precursors. DEMs were analyzed on a case-by-case manner. From initially 157 DEMs, 124 disconnected metabolites (along with 58 reactions) were excluded from the models: 14 DEMs came from 7 spontaneous reactions; 59 were carbohydrate substrates derived from wide range transport reactions and were not used by any other reaction in the network; 51 were excluded as they did not interfere with the overall metabolism and/or the enzyme had already been assigned to one or many other reactions. Orphan reactions were excluded unless they were essential for biomass growth. All excluded reactions (non-metabolic, generic, duplicated, orphan) and all DEMs (validated and excluded) are listed in Additional file 1: Tables S2c and S2d. It is interesting to point out that other unknown enzymes (or even *moonlight* enzymes) from these organisms may indeed use some of the excluded DEMs; however, since we have no experimental evidence at present, we could not assess their interference in the metabolic models.

The remaining DEMs consisted mainly of cofactors and biomass precursors (such as nucleotides, amino acids, fatty acids) disconnected from the rest of the network. They were solved along with the biomass precursor check; 141 reactions were added to allow growth: 1 biomass reaction, 14 enzymatic reactions, 1 drain-synthetic reaction, 105 transport reactions and 5 spontaneous reactions. Transport reactions were considered as such even if an enzymatic activity was also present (i.e. the import of sugars with a concomitant phosphorylation of substrate). In order to correctly assign possible and specific transporters, we performed an extensive literature search and reciprocal BLAST alignments to characterized transporters in other species. These results have to be experimentally confirmed, since assignment of the correct substrate based only on sequence homology remains an open problem in genomic annotation.

**Fig. 1:**
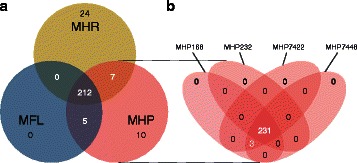
Venn diagrams representing the comparison of refined networks. Numbers represent the exclusive and common reactions present in the refined networks (**a**) between species, and (**b**) between selected strains of *M. hyopneumoniae*. This analysis shows that most of the metabolism is common to all organisms. MHR: *M. hyorhinis*; MHP: *M. hyopneumoniae*; MFL: *M. flocculare*

We also changed inconsistencies of reversibility and cofactor usage. Even after all efforts, four DEMs still remained: TTP, hexulose-6-phosphate, deoxyinosine and xanthosine-5-phosphate. After the final addition of 101 exchange reactions, we ran FBA tests to check the consistency of all models. If a reaction was essential for biomass growth and no homolog gene was found in the genome, an orphan reaction was validated or added to the reconstruction for modeling reasons only. The resulting refined pan-network had a total of 457 reactions and 258 GPR associations. A comparison between the species models may be found in Fig. 1. These results indicate that all strains from all species are indeed metabolically similar.

The overall characteristics of each reconstruction can be seen in Table 2. A list of all reactions and metabolites added to the models along with the corresponding genes can be seen in Additional file 1: Table S2.

**Table 2:** Characteristics of reconstructed models from different strains and species

Organism	Model name	Number of genes in model	Number of GPRs ∗^*a*^	Enzymatic reactions ∗^*b*^	Transport reactions ∗^*b*^	Spontaneous reactions ∗^*c*^	Total number of reactions ∗^*d*^
MHP_232	iMF170	170	233	208	111	9	426
MHP_168	iMF172a	172	234	209	111	9	427
MHP_168L	iMF172b	172	234	209	111	9	427
MHP_7422	iMF168	168	234	209	111	9	427
MHP_7448	iMF171	171	234	209	111	9	427
MHP_J	iMF172c	172	234	209	111	9	427
MHP	iMFmhp	NA ∗^*e*^	234	209	111	9	427
MHR_HUB1	iMF177	177	239	209	111	6	423
MHR_GDL1	iMF175	175	242	210	113	6	426
MHR_SK76	iMF181	181	243	211	113	6	427
MHR_17981	iMF182	182	243	211	113	6	427
MHR	iMFmhr	NA ∗^*e*^	243	211	113	6	427
MFL_27399	iMF159	159	217	196	105	9	401
MFL_27716	iMF157	157	217	196	105	9	401
MFL	iMFmfl	NA ∗^*e*^	217	196	105	9	401
Pan-Network	iMFpan	NA ∗^*e*^	258	230	117	9	457

Notes:

∗^*a*^: GPRs are gene-proteins-reaction associations present in each genome. For iMFmhp, iMFmhr, iMFmfl and iMFpan that account for more than one species, we added a GPR to the species model when any of the strains harbored a gene responsible for a specific activity

∗^*b*^: Reactions were considered as transport reactions even if the transporter was capable of performing a concomitant enzymatic activity

∗^*c*^: Spontaneous reactions included diffusion of small molecules and spontaneous conversions

∗^*d*^: Total number of reactions included also all exchange reactions needed for the mathematical modeling

∗^*e*^: Genes in the pan-network and species models were added synthetically (one per reaction), to enable a reaction essentiality analysis

### Biomass composition and biomass equation

The biomass equation drained all precursors (in their molar biological ratios) into biomass. Biomass composition according to the “Methods” section along with a detailed description of the assembly of the biomass reaction can be found in Additional file 1: Table S3 and Additional file 2. Considering the percentage contribution of each of the components to the overall cell, an approximate biomass elementary composition for *M. hyorhinis* was computed as follows: *CH*_1.57_*O*_0.36_*N*_0.21_*P*_0.02_*S*_0.02_, with traces of calcium, chlorum, cobalt, copper, iron, potassium, magnesium, manganese, molibdenium and zync and molecular weight (MW) on a C-mole basis of 23.89 *g*/ *C*−*mol*. We had a slightly different composition for *M. hyopneumoniae* and *M. flocculare*: *CH*_1.59_*O*_0.34_*N*_0.21_*P*_0.16_*S*_0.02_, with the previously mentioned trace compounds and MW on a C-mole basis of 23.31 *g*/ *C*−*mol*.

### Metabolism of *M. hyorhinis*, *M. hyopneumoniae* and *M. flocculare*

**Fig. 2:**
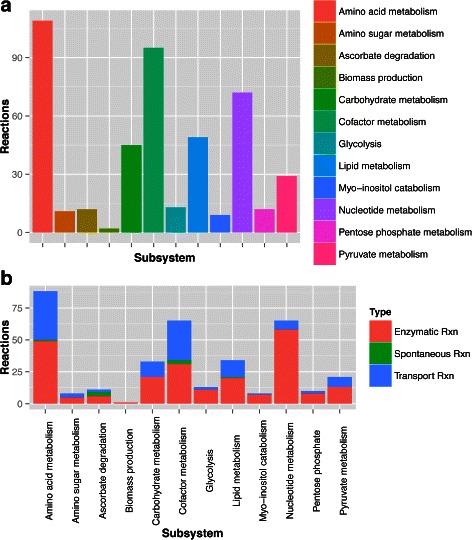
Distribution of the model reactions in the subsystems. The 457 reactions present in the model iMFpan were separated into (**a**) biological subsystems and (**b**) further into reaction types, with the exclusion of exchange reactions in this analysis

**Fig. 3:**
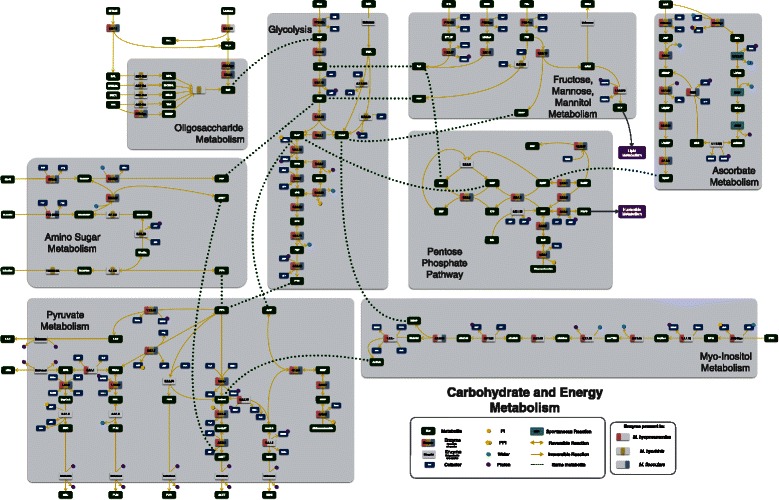
Global energy and carbohydrate complete models. Metabolites are depicted in *dark green* and separate enzymatic activities for *M. hyorhinis*, *M. hyopneumoniae* and *M. flocculare* can be seen in *yellow*, *pink* and *blue*, respectively. Whenever an enzyme is missing from the three species, the enzyme rectangle is depicted in *grey*. Complete list of metabolite abbreviations and EC numbers can be found in Additional file 4

**Fig. 4:**
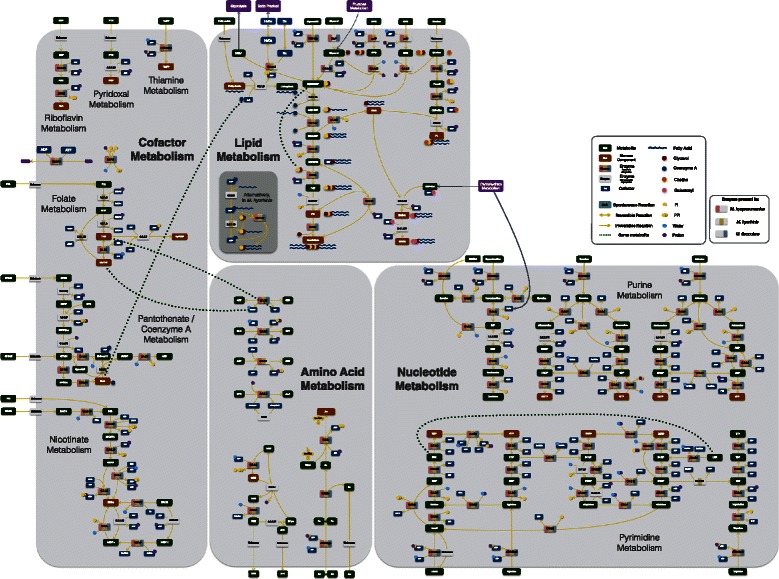
Lipid, amino acid, nucleotide and cofactor complete models. Metabolites are depicted in *dark green* and separate enzymatic activities for *M. hyorhinis*, *M. hyopneumoniae* and *M. flocculare* can be seen in *yellow*, *pink* and *blue*, respectively. Whenever an enzyme is missing from the three species, the enzyme rectangle is depicted in *grey*. Complete list of metabolite abbreviations and EC numbers can be found in Additional file 4

Similar to most *Mycoplasma* species studied so far [46–48], all reconstructed networks exhibit low connectivity due to the simplicity of the biological model. Out of the 457 reactions in the final pan-network, 258 had in at least one species a GPR association. From these, 212 were common to all species (Fig. 1). The overall metabolism from the models reconstructed consisted of 11 distinct subsystems: amino sugar metabolism, amino acid metabolism, carbohydrate metabolism (further broken down into: glycolysis, pentose phosphate pathway, ascorbate degradation, myo-inositol degradation, general carbohydrate metabolism, and pyruvate metabolism), cofactor metabolism, lipid metabolism, and nucleotide metabolism (Fig. 2 for pan-network; the number of GPR associations for each species can be seen in Additional file 1: Tables S4a and S4b). While all enzymes were present in glycolysis, most metabolic pathways had major enzyme gaps. In the product and cofactor metabolism, for instance, gaps accounted for up to 50 % of the reactions. *M. flocculare* was the only species that did not show any exclusive metabolic activities in the models, although this could not be verified in vivo due to lack of biochemical studies for this species. *M. hyopneumoniae* had 10 exclusive reactions, linked to the myo-inositol metabolism and alcohol dehydrogenase activity. *M. hyorhinis* had 24 exclusive reactions; most of them corresponded to carbohydrate metabolism. A global model comparing each species enzymatic capabilities can be seen in Figs. 3 and 4. The complete list of metabolite abbreviations and EC numbers for these two figures can be found in Additional file 3.

#### Transporters

Transporter assignment was made *in silico* based on sequence similarity to known transporters. We were able to identify in all species complete putative ABC transporters for multidrug/toxin efflux and for import of sugars, oligopeptides, peptides, spermidine/putrescine, phosphonate/phosphate/thiamine, cobalt, manganese/zinc and glycerol. In the search of a possible nucleotide transporter, we came across with an unpublished work from Lee [68] in which a system previously annotated as sugar ABC transporter was experimentally validated as involved in nucleoside uptake in *Mycoplasma bovis*. These results may be highly speculative, and need further verification, but we were able to find homolog genes in the genomes of all species. *M. hyopneumoniae* seems to have an extra ABC transporter proposed for myo-inositol and *M. hyorhinis* has a unique predicted ABC transporter for maltose/maltodextrins. Three complete phospho-transferase transport systems (PTS) were common to all species. Although specificity of PTS is difficult to ascertain, we have tried to compare conserved domains from validated PTS substrate-specific enzymes II (available in TransportDB [69]) and candidates from the three species. Genes coding enzyme I and phosphocarrier were found in all strains, except in MHR_HUB1. From sequence similarity and gene context we were able to annotate the correct gene. Based on these *in silico* predictions from domain retrieval, we propose the following: one PTS non-specific for sugar, one with specificity for fructose and another for mannitol. *M. hyopneumoniae* and *M. flocculare* also share two extra complete systems: one possibly for ascorbate and one for N-acetylglucosamine (GlcNAc). *M. hyorhinis* and *M. hyopneumoniae* seem to have an extra IIB component specific for glucose, and along with the other components of the general sugar PTS were proposed to form a complete glucose PTS.

The protein GlpU, coded by gene MPN241 in *M. pneumoniae*, was recently described to act on the uptake of glycerophosphodiesters [70]. We found homologs of this gene in all strains of *M. hyopneumoniae* and *M. hyorhinis* adjacent to the gene coding for the enzyme responsible for the metabolization of these substrates (GlpQ) [71]. In MHR_HUB1, this gene was annotated as a pseudo gene, but from gene context, we were able to validate it as GlpU. All species also possess several unspecific amino acid permeases, a glycerol facilitator protein (GlyF), which is thought to be less efficient than the glycerol ABC transporter (ABC-Gly) [35]) and a major facilitator protein (MIF) with unknown specificity. We could also find common transporters for cobalt and magnesium, chromate, magnesium, potassium, zinc and a cation ATPase. *M. hyorhinis* has two extra transport systems: one for sialic acid (also known as N-acetyl neuraminate) and one sodium/phosphate co-transporter. Details of the transport search throughout all species is summarized in Additional file 4.

#### Carbohydrate and energy metabolism

##### Glycolysis

As previously described in [18], all genes for the glycolysis exist in all strains of all species. *M. hyorhinis* may be able to convert dihydroxyacetone (DHA) directly to dihydroxyacetone-phosphate (DHAP) with the action of DHA kinase (2.7.1.29), but the mechanism of DHA uptake is not clear.

##### Pentose Phosphate

In agreement with previously described literature [14, 18, 72], we found no enzymes for the oxidative branch of the pentose phosphate pathway in the studied species, turning ribulose-5-phosphate into a DEM. This problem was overcome by the assumption that the reaction ribulose-phosphate-3-epimerase (EC 5.1.3.1) can be reversible, as reported in *Lactobacillus casei* [73]. Out of the 11 reactions present in the final models, we found homologs for 9 in all species. Although a gene coding for transaldolase (EC 2.2.1.2) is missing in all species, we included this reaction in the models to prevent both sedoheptulose-7-phosphate and erithrose-4-phosphate from becoming DEM.

##### Ascorbate Metabolism

Only one enzyme is missing from the typical pathway: L-ascorbate-6-phosphate-lactonase (UlaG). This enzyme is responsible for the turnover of L-ascorbate-6-phosphate to 3-keto-gulonate-6-phosphate (EC 3.1.1.-). From the gene context, MHP_7448_0377 from MHP_7448 and its homologs from all other organisms may encode the missing enzyme. The gene was annotated as a conserved hypothetical protein with a proposed phosphotriesterase activity.

##### Uptake of other carbohydrates

While the uptake of fructose, mannitol and mannose is similar in all species and they all can be fed into glycolysis as fructose-6-phosphate (F6P) and DHAP, *M. hyorhinis* has an extra set of enzymes enabling the conversion of fructose-1-phosphate into fructose-1,6-biphosphate by the action of 1-phosphofructokinase (EC 2.7.1.56). Only in *M. hyopneumoniae* the conversion of glyceraldehyde into glycerol might be possible through the moonlight activity of alcohol dehydrogenase (EC 1.1.1.372), although up to date no experimental evidence can sustain that assumption. The presence of a unique transcriptional unit (TU) with carbohydrate metabolism-related genes in *M. hyorhinis* probably enables the uptake and metabolization of isomaltose (EC 3.2.1.10), maltose (EC 2.4.1.8), trehalose (EC 2.4.1.64 and EC 3.2.1.93) and sucrose (EC 3.2.1.48) into either glucose-6-phosphate or F6P. This may be related to the fact that *M. hyorhinis* can overgrow the other two species in several growth media.

##### Myo-inositol metabolism

A TU for the myo-inositol catabolism is present in all *M. hyopneumoniae* strains, with the exception of the enzyme 5-dehydro-2-deoxy-phosphogluconate aldolase (IolJ, EC 4.1.2.29). The gene encoding this enzyme in other organisms is similar to the fructose-biphosphate aldolase (Fba) from glycolysis (EC 4.1.2.13). Since there are two copies annotated for Fba in *M. hyopneumoniae*, we proposed one of them as candidate for this activity based on sequence alignments with both genes from *Bacillus subtilis*. Inositol can be used as a carbon source and also produces acetyl coenzyme-A (AcCoA), which can be a source of cofactor coenzyme-A (CoA).

##### Amino sugar metabolism

*M. hyopneumoniae* and *M. flocculare* models could uptake and convert GlcNAc to N-acetyl-glucosamine-6-phosphate (GlcNAc6P); *M. hyorhinis* models imported sialic acid and converted it to GlcNAc6P in three steps that are unique to *M. hyorhinis* (EC 4.1.3.3, EC 2.7.1.60, EC 5.1.3.9). At first, the genes coding for these three enzymes were absent from MHR_HUB1 and MHR_GDL1. However, based on chromosome alignment and synteny with other genes, we were able to find the regions and correctly annotate all genes.

##### Pyruvate metabolism

Out of fifteen enzymatic reactions in this pathway, only three were not found in any organism. Conversion of pyruvate to lactate and acetate were possible in all species. From literature data, we knew that *M. hyopneumoniae* was able to produce oxaloacetate, malate and 2-oxoglutarate (2KG) [74, 75]. Decarboxylation of 2KG to succinyl coenzyme-A (SucCoA) has been previously reported in *M. hyorhinis* extracts [76]. Although we did not find any candidate for the activity of aspartate transaminase (EC 2.6.1.1), gap-filling proposed that this activity could be performed by the pyruvate dehydrogenase complex. Moreover, Cordwell and collaborators [77] suggested that in *Mollicutes* lactate dehydrogenase (Ldh, EC 1.1.1.27) could also function as malate dehydrogenase (Mdh, EC 1.1.1.37). *M. hyopneumoniae* is the only organism among the three to have a gene coding for an alcohol dehydrogenase, probably enabling the turnover of AcCoA to acetaldehyde (EC 1.2.1.1 0) and ethanol (EC 1.1.1.1). Conversion of pyruvate to formate by pyruvate formate-lyase (EC 2.3.1.54), malate to fumarate by fumarate hydratase (EC 4.2.1.2) and SucCoA to succinate by succinyl-CoA synthetase (EC 6.2.1.5) were added to the models in a final step to accommodate the results from the metabolomics experiments.

#### Lipid metabolism

The lipid metabolism in the models of the three species included the uptake of glycerol, choline, glycerophosphodiesters and fatty acids to the production of the biomass precursors cardiolipin, 1,2-diacyl-sn-glycerol, phosphatidylglycerol, phosphatidylcholine and galactosyl-diacylglycerols (except in *M. hyorhinis*). Extracellular cholesterol and sphyngomyelin were incorporated unmodified directly into biomass. While *M. hyopneumoniae* seems to have four different ways to uptake and metabolize glycerol (ABC-Gly, GlyF, GlpU and directly from glyceraldehyde) *M. hyorhinis* lacks one and *M. flocculare* lacks two of them (Fig. 4). The turnover of glycerol-3-phosphate into DHAP by the action of glycerol-3-phosphate oxidase (GlpO, EC 1.1.3.21) allows the usage of glycerol as the sole carbon source, with the production of highly toxic hydrogen peroxide. This was only possible in the *M. hyorhinis* and *M. hyopneumoniae* models.

The enzyme responsible for the turnover of glycerol-3-phosphate into acyl-sn-glycerol-3-phosphate (glycerol-3-phosphate 1-O-acyltransferase, EC 2.3.1.15) was found only in the genomes of *M. hyorhinis*. Although this can be seen as a possible difference between the species, we had to add the reaction to the models from the other species to enable growth. It is possible that the other two species import this metabolite directly from the media, however further experiments are required to confirm this hypothesis. No homologs for the enzyme that produces phosphatidylglycerol (phosphatidylglycerophosphatase, EC 3.1.3.27) were found; however, all *Mollicutes* definitely synthesize this metabolite, and hence must use a hitherto undetected enzyme for this step [78]. The acyl-carrier protein (ACP) was only found in the genomes of *M. hyorhinis* and may act as a fatty acid and CoA donor in these organisms. Orphan reactions for the production of glycolipids (EC 2.4.1.46 and EC 2.4.1.241) were added to models for *M. hyopneumoniae* and*M. flocculare* based on the presence of these metabolites in vivo [56].

#### Amino acid metabolism

The import of amino acids was added in two forms: oligopeptide import and cleavage or single amino acid import. tRNA charging accounts for most of the reactions in this pathway (23 reactions). Production of the biomass precursor S-adenosyl-methionine from methionine is possible in all strains (methionine adenosyltransferase, EC 2.5.1.5). Moreover, since the enzyme adenosylhomocysteine nucleosidase (EC 3.2.2.9) is present in all species, we added two reactions to restore connectivity between these two subpathways (EC 2.1.1.- and EC 4.4.1.21). This addition resulted in the production of two metabolites essential for quorum sensing and cell communication in other species: S-rybosyl-homocysteine and autoinducer-2.

#### Nucleotide metabolism

The mycoplasmas in this study cannot synthesize *de novo* purines and pyrimidines; therefore, they have only salvage pathways and interconversions to supply the cell with nucleic acid precursors. The three species have the same enzymatic capabilities, except for the presence of thymidylate synthetase (EC 2.1.1.46) in *M. hyorhinis*, which is also important for cofactor metabolism and is responsible for the conversion of dUMP and 5,10-methylene-tetrahydrofolate (MeTHF) to dTMP and dihydrofolate. Overall, the nucleotide metabolism consists in the uptake of guanine, adenine, uracil, thymine and cytidine and produces all deoxy-ribonucleotides (dATP, dCTP, dGTP, TTP) and ribonucleotides (ATP, CTP, GTP and UTP).

#### Cofactor metabolism

Around 60 % of the enzymes that comprise the cofactor metabolism of the reconstructed models were not found in any species. For instance, thiamine-pyrophosphate is imported unchanged directly into biomass, while pyridoxal is imported and converted to the cofactors pyridoxal-phosphate by the action of pyridoxal kinase (EC 2.7.1.36 missing in all species). From the pantothenate/coenzyme-A metabolism, we only found homologs for the conversion of 4’-phosphopantetheine into dephospho-coenzyme-A by pantetheine-phosphate adenylyltransferase (EC 2.7.7.3). Three forms of folate are incorporated to the biomass: MeTHF, 5,6,7,8-tetra-hydrofolate and 10-formyl-tetra-hydrofolate. We assumed that folate was imported and converted to these three biomass cofactors by: dihydrofolate reductase (EC 1.5.1.3, only present in *M. hyorhinis*), serine hydroxymethyltransferase (EC 2.1.2.1, present in all species) and formate-tetrahydrofolate ligase (EC 6.3.4.3, missing in all species). This may also account for the better fitness of *M. hyorhinis* when compared to the other two species. Although no homologs for NAD kinase were found, two reactions were added (EC 2.7.1.23 and EC 2.7.1.86) to allow the presence of NADP and NADPH, which are cofactors for several essential reactions. The NADPH and NADH produced from the degradation of glucose and other carbohydrates are recycled by the action of two enzymes: NADH oxidase (hydrogen peroxide forming, EC 1.6.3.3) and thioredoxin disulfide-reductase (EC 1.8.1.9).

### Mycoplasmas cultivation

**Fig. 5:**
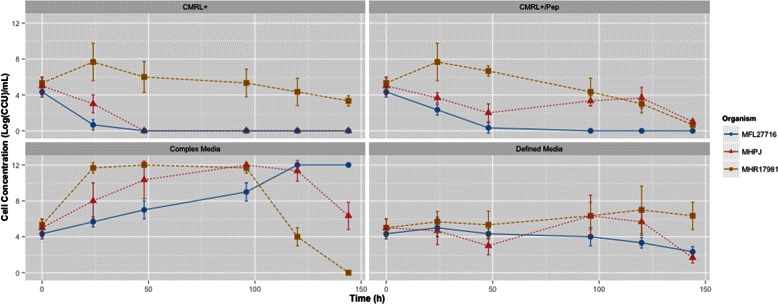
Cultivation curves in defined and complex media by species. Cell concentrations were estimated by the CCU method and error bars were calculated as the standard deviation between triplicate time-matched samples. As expected, the three species had better growth rates in the complex medium than in defined media. *M. hyopneumoniae* strain J (MHP); *M. flocculare* strain 27716 (MFL); *M. hyorhinis* strain ATCC17981 (MHR)

We cultivated the three species of mycoplasmas in complex and defined media to gather experimental information and compare to *in silico* growth (Fig. 5). In the CMRL+ medium, *M. hyopneumoniae* and *M. flocculare* remained viable only when peptone was present (CMRL+/Pep). The presence of peptone appeared to have a negative effect on *M. hyorhinis* growth, but more tests should be performed to verify this hypothesis. Yus+ medium did not allow proliferation in any species, but maintained cell concentration and viability even after 5 days of culture (if inoculated afterwards in complex media). These results are in agreement with a previous work performed by Bertin and colleagues [79] in *M. mycoides* subsp. *mycoides* that shows that CMRL-1066 contains all components to support cellular metabolism but not growth. Here, the supplemented versions CMRL+ or CMRL+/Pep allowed proliferation only for *M. hyorhinis* during the first 24 h of growth. It seems that after this period of time, one or more essential metabolites initially present were no longer available. By comparing the models reconstructed in this work with the media composition tested for the three species, it might seem plausible that key cofactors might not be delivered to mycoplasmas in the correct form. For example, thiamine pyrophosphate should be directly delivered to all species instead of its precursor thiamine, and pyridoxal-5-phosphate might be the cofactor of choice for the media composition in place of pyridoxal. A list of predicted biomass precursors from the models *versus* the actual precursors found in the Yus+ medium can be seen in Additional file 1: Table S5. This new proposed defined medium will be submitted in the near future to cultivation tests with *M. hyorhinis*, *M. hyopneumoniae* and *M. flocculare*.

### Metabolomics of swine mycoplasma

NMR analysis of the culture media was performed in order to detect possible differences in the metabolism of the three species. All data measured in defined and complex media can be seen in Additional file 1: Tables S6a and S6b.

**Fig. 6:**
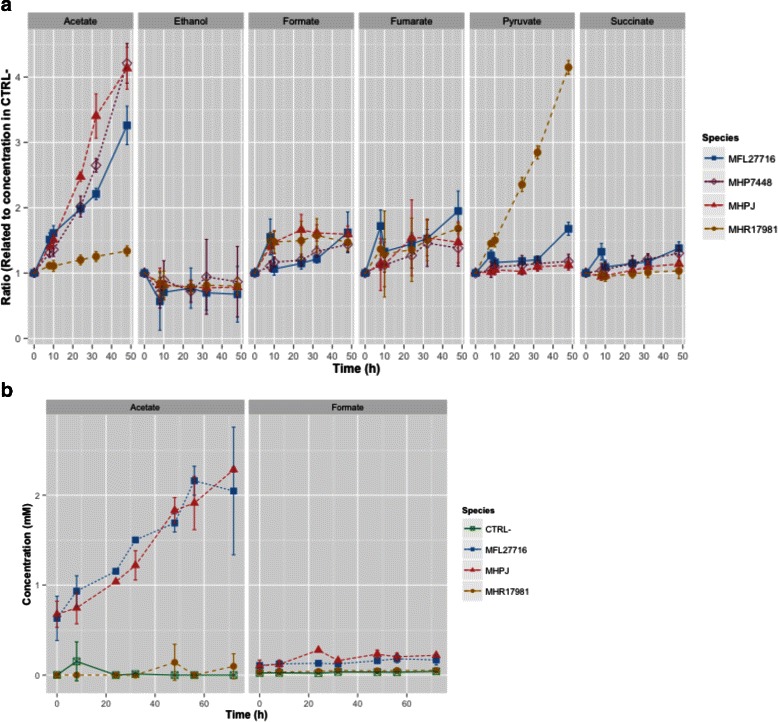
Distinct products of the metabolism of pyruvate from growth in complex Friis medium and Yus+ medium of *M. hyopneumoniae* strains 7448 (MHP_7448) and J (MHP_J), *M. flocculare* strain 27716 (MFL_27716) and *M. hyorhinis* strain ATCC17981 (MHR_17981). In complex medium, we calculated the ratio between the peak signal in cultivated versus control medium and error bars were calculated as the standard deviation between triplicate time-matched samples. For defined medium, we detected the actual concentration for the metabolites and error bars were calculated as the standard deviation between duplicate time-matched samples. **a** In complex medium, *M. hyopneumoniae* (both strains) and *M. flocculare* can produce high amounts of acetate; the yields are even higher from *M. hyopneumoniae*. *M. hyorhinis*, on the other hand, produces low concentrations of acetate in this medium. The final glycolysis product for *M. hyorhinis* is thus pyruvate. **b** In defined medium, *M. hyopneumoniae* (strain J) and *M. flocculare* produce similar amounts of acetate while *M. hyorhinis* contains only residual levels of this metabolite. The three species can produce low amounts of formate in both media

The major differences in both complex and defined media were related to the metabolism of pyruvate (Fig. 6): *M. hyorhinis* produced higher quantities of pyruvate at the end of 48 h in complex medium. As a result, pyruvate conversion to acetate was detected in low quantities for this species in both media. *M. hyopneumoniae* and *M. flocculare*, on the other hand, produced higher amounts of acetate (this was even more pronounced for the growth of *M. hyopneumoniae* in complex medium). Low amounts of formate, fumarate and succinate were also detected, and this indicates the presence of genes encoding the enzymes pyruvate formate-lyase (EC 2.3.1.54), fumarate hydratase (EC 4.2.1.2) and succinyl-CoA synthetase (EC 6.2.1.5) in these genomes. As previously mentioned, all models were thus modified to accommodate these activities. Formate production seems to be independent from acetate production in *M. hyopneumoniae* and *M. flocculare*. However, the similar low concentrations of acetate and formate in both growth media from *M. hyorhinis* might be an indication that both are produced concomitantly by the action of pyruvate formate-lyase (EC 2.3.1.54), phosphate acetyl-trasnferase (EC 2.3.1.8) and acetate kinase (EC 2.7.2.1).

To check if the lower concentrations of acetate were a result of an impaired activity of the pyruvate dehydrogenase complex in *M. hyorhinis*, we compared the predicted proteins PdhA, PdhB, PdhC and PdhD from all species. The known active sites of ODP2 (homolog of PdhC) and OLDH1 (homologs to PdhC and PdhD respectively) from *Bacillus subtilis* were analyzed in search for a possible mutation. No active sites have been described for PdhA and PdhB so far, but the enzymes for all three swine mycoplasmas do not present significant differences. Although not all active sites from PdhD seem to exist in mycoplasmas, all predicted proteins from the species analyzed (including *M. genitalium* and *M. pneumoniae*) are similar in terms of active sites, when these are present. The only difference between *M. hyorhinis* and the other two species was found in PdhC, in the protein portion related to the binding to PdhA and PdhD. This portion is the most variable between the species. An enzyme characterization should be made in order to verify if this complex is not active in this species, or if it is responsible for another unforeseen activity.

The presence of myo-inositol in swine serum [80] might be directly related to the higher production of acetate in the growth of *M. hyopneumoniae* in complex medium, if compared to *M. flocculare*. Since the myo-inositol pathway produces AcCoA, the recovery of a molecule of CoA from myo-inositol is possible and may be directly linked to the production of acetate. Since the myo-inositol catabolic pathway is one of the few distinctions between the metabolic models of *M. hyopneumoniae* and *M. flocculare*, it is possible to assume that the differences in acetate concentration in vitro may indeed arise from the ability of *M. hyopneumoniae* to uptake myo-inositol. Indeed, when no source of myo-inositol is present (defined medium), no difference in the concentration of acetate is observed for these two species.

The analysis of the amino acid uptake was not trivial since both media contained peptone, which at first was not measured through the CPMG experiment but, in the course of time, was degraded into single amino acids, and thus changed the overall signal of the NMR spectra. To address this issue, we only took into account the Yus+ medium at a single time point after 72 h of growth. *M. hyorhinis* seemed to have lower concentrations of most amino acids at the end of the growth curve if compared either to the control medium or to the other species. This may be related to several factors, such as lower rates (in comparison to the other two species) of peptone degradation by membrane proteases or higher uptake rates of amino acids by *M. hyorhinis*. At this point, we could no longer verify which hypothesis is more suitable for this particular distinction, however, since peptone was not necessary for the maintenance of the viability of *M. hyorhinis* in the defined medium, it is possible that this species may harbor transporters with higher specificity or effectiveness for single amino acids.

The results as concerns the other metabolites indicate for the three species the uptake of glucose and nucleotides.

### Flux balance analysis (FBA)

FBA was used to check the properties and capabilities to produce detected metabolites by NMR spectra of all models (also during the reconstruction and refinement process). FBA simulations were performed with an *in silico* medium that contained all biomass precursors. Similarly to the in vitro growth results, in the presence of oxygen, acetate was the main product and NADH was recycled by the conversion of molecular oxygen into water. In the absence of oxygen (not tested in vitro), all models were able to grow but the main product became lactate. As expected, the availability of myo-inositol in the *in silico* medium allowed an alternative source of CoA and higher concentrations of acetate as a final product in the models for *M. hyopneumoniae*. Hydrogen peroxide production was linked to the metabolism of glycerol in both *M. hyopneumoniae* and *M. hyorhinis*, while the models for *M. flocculare* did not produce toxic levels of this metabolite. Since we have no information on gene essentiality for any species, we checked reaction essentiality in the pan-network. This means that we deleted individual reactions (all transport, enzymatic and spontaneous reactions) even if they did not have a GPR association. A total of 111 reactions (69 enzymatic, 37 transport, and 5 spontaneous reactions) were essential for growth in the pan-network (Additional file 1: Table S7).

## Discussion

In this work, we created a metabolism as realistic as possible for the three known mycoplasmas present in the respiratory tract of swine. It is essential to point out that, although we had to include 30 % of orphan reactions in order to allow growth, we only used about half of the genomes of these organisms. The other half consists either of hypothetical or of conserved hypothetical proteins [18], which may in part fill the missing gaps of the models. The number of essential reactions in the pan-network in this work is not directly comparable to the number of indispensable genes predicted for *M. genitalium* (382 genes) or *M. pneumoniae* (310 genes) [81, 82]. Gene essentiality accounts for more than metabolic enzymes which means that proteins not included in our models, related to protein synthesis, DNA polymerization or RNA turnover, were not accounted for in the essential reactions of our models. If we added these proteins, we would possibly arrive at numbers closer to those of *M. genitalium* and *M. pneumoniae*. The lack of experimental information on gene essentiality is also a setback for the validation of the models created here. Further experiments on this matter should help us better refine and complete the networks.

**Fig. 7:**
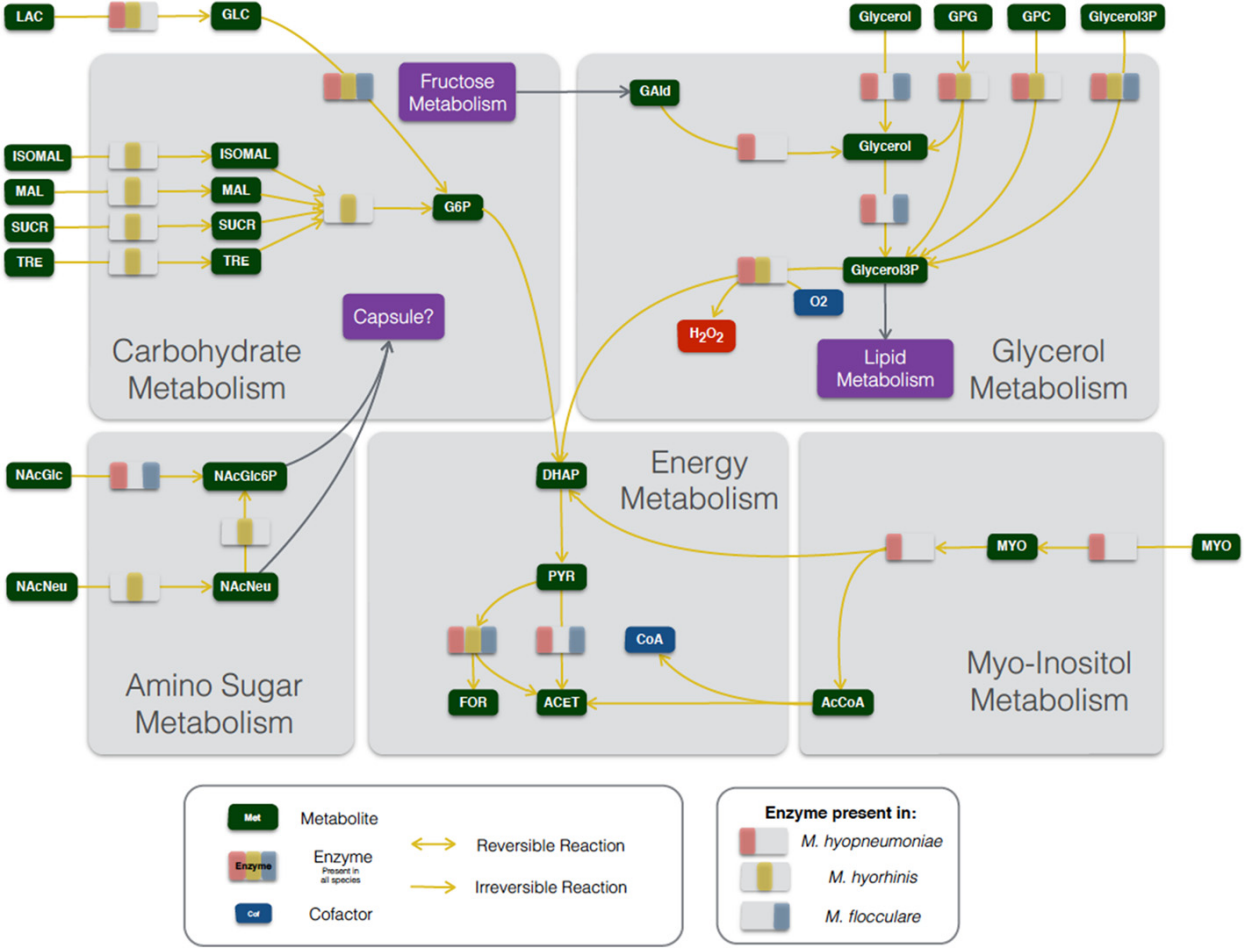
Differential metabolism of *M. hyorhinis*, *M. hyopneumoniae* and *M. flocculare*. Metabolites are depicted in *dark green* and separate enzymatic activities for *M. hyorhinis*, *M. hyopneumoniae* and *M. flocculare* can be seen in *yellow*, *pink* and *blue*, respectively. Whenever an enzyme is missing from the three species, the enzyme rectangle is depicted in *grey*. Complete list of metabolite abbreviations and EC numbers can be found in Additional file 4

As for most of the *Mycoplasma* species studied [46–48], all reconstructed networks exhibit low connectivity due to the simplicity of the biological model. We were able to show in this work that the three swine mycoplasma species have similar metabolic capabilities, except for the metabolism of myo-inositol, amino sugar, carbohydrates and glycerol (Fig. 7). Overall, the methods used here enabled us to address some of the main problems caused by most automatic reconstruction methods which are the permissive inclusion of pathways and over prediction of capabilities. Annotation errors arise with the attribution of ambiguous or partial EC numbers [83, 84] and the propagation of these errors may then lead to many other ones. Thus, it is known that the prediction of GPR associations based only on name-matching and EC-codes is not sufficient to add confidence to a model [84]. When we decided to simultaneously refine the 12 models, we enhanced the confidence of each GPR association, by adding information on the synteny between the genomes, protein sequence alignments and phylogenetic distance between orthologs.

Our main objective was not only to reconstruct the metabolic models for *M. hyorhinis*, *M. hyopneumoniae* and *M. flocculare*. We also wanted to compare the metabolism of the three species and find possible links to virulence, host-colonization capacity and life-style. *M. hyorhinis*, for instance, is not only a pathogen, but also a common contaminant in many mammalian cell cultures [85]. This may be explained by the wide range of carbohydrates which this organism can uptake and metabolize. This may be a crucial factor to the ability of *M. hyorhinis* to grow in diverse sites inside the host [7] and even to invade other hosts and potentially develop cancer in humans [58, 86]. *M. hyopneumoniae* has been detected in other sites such as brain, liver and spleen [87, 88], but *M. flocculare* has never been detected outside of the respiratory tract of swine.

One may wonder what could explain this if both organisms are so similar. *M. hyopneumoniae* is up to date the only *Mycoplasma* species with sequenced genome that has the genes for the catabolism of myo-inositol. Myo-inositol is readily abundant in the bloodstream of mammalian hosts, and can be used as a secondary carbon source for energy production [89]. *Mycoplasma iguanae* has been described to produce acid from inositol [90]. However, there is no available complete genome sequence for this organism, making impossible any comparison of the genes involved in this pathway. Although at this point we cannot confirm that this pathway is functional in *M. hyopneumoniae*, the NMR results we obtained suggest that myo-inositol might be directly related to the higher production of acetate in complex medium of *M. hyopneumoniae* if compared to *M. flocculare*. This assumption is based on two factors: first, a previous work has detected myo-inositol in swine serum, which is a component of the complex medium [80]; and second, the only difference in the networks that could influence the acetate concentration is the myo-inositol catabolism. This pathway may also be involved with an alternative production of AcCoA, precursor to CoA, an essential cofactor for growth in all species. These metabolic distinctions may help *M. hyopneumoniae* to grow in diverse sites if compared to *M. flocculare*. This is particularly interesting when we take a close look into the composition of lung surfactant from several mammalian models [91], which likely contains myo-inositol as a degradation product from phosphatidylinositol. Thus, it is possible that the myo-inositol catabolism in *M. hyopneumoniae* is one of the reasons for the high virulence of this species when compared to *M. flocculare* and *M. hyorhinis*.

A striking result from the NMR results was the reduced capability of *M. hyorhinis* to produce acetate. This was not foreseen in the model reconstruction, but predicted protein sequences from the pyruvate dehydrogenase complex of the three species showed a particular distinction in PdhC, more specifically in the region responsible for binding to other complex components. We could not verify at this point if this complex is not active in the particular growth conditions of our work in *M. hyorhinis* or if it is responsible for another unforeseen activity. However, a transcriptome profiling of *M. hyorhinis* has detected both pdhA and pdhB in the pool of genes with the highest number of transcript reads [32], indicating that the complex might be translated in vivo. Moreover, pyruvate dehydrogenase activity has been previously detected in *M. hyorhinis* extracts [75], and it seems to be correlated to oxygen availability. In our cultivations, the cells are not grown in a complete aerobic system, which may explain the differences between our findings and those previously published.

Cultivation tests in vitro also showed that none of the species was able to grow in none of the defined media. This is probably due to a lack of the correct cofactors as previously mentioned. For instance, thiamine, pyridoxal/pyridoxine, pantothenate, spermine and folate were the actual media components tested, but the reconstructed models were not able to convert them to biomass precursors (thiamine pyrophosphate, pyridoxal-5-phosphate, 4-phospho-pantheteine, spermidine and THF). Only *M. hyorhinis* seems to be able to uptake folate directly while the other two species might need intermediate metabolites. This might also explain why *M. hyorhinis* is extensively found as a cell culture contaminant. Even though we supplemented CMRL-1066 (CMRL+) with all missing components present in the Yus+ medium, cells maintained viability for longer periods in the latter. Cofactors present originally in both media existed in lower concentrations in the CMRL-1066. This means that possibly one or more cofactors with higher concentrations in the Yus+ medium are essential for the viability of the three species. Common cofactors with higher concentration in Yus+ medium included: choline, folate, pantothenate, pyridoxal, thiamine and spermine.

Other differences among the species seem to indicate that *M. hyopneumoniae* and *M. flocculare* lack one or more amino acid transporters. This may be due to two things: (i) both remained viable for longer periods whenever CMRL+ was supplemented with peptone, and (ii) we detected a higher amino acid accumulation in the defined media if compared to *M. hyorhinis*. *M. hyorhinis* on the other hand may have all the transporters for single amino acids, but this hypothesis needs to be further verified. From sequence analyses, we did not find significant differences among the species that would explain this distinct behavior. Moreover, since *M. hyorhinis* seemed to overgrow in the first 24 h of culture in both CMRL media (CMRL+ and CMRL+/Pep), it is possible that one or more compounds were missing in the Yus+ medium in order to allow growth of this species. Possible candidates for such (present in CMRL-1066 and absent in the Yus+ medium) would be: CoA, nicotinamide, ascorbate and/or pyridoxine.

It is still not clear if any of the swine mycoplasmas studied here indeed have a polysaccharide capsule, given that only one study has assesed its presence in *M. hyopneumoniae*. However, this is particularly important because in this study Tajima and Yagihashi [28], as discussed before, reported that capsular polysaccharides from *M. hyopneumoniae* play a key role in the interaction between pathogen and host. Moreover, it has been reported that some strains of *M. hyopneumoniae* become less pathogenic in broth culture and, after serial passages, lose their ability to produce gross pneumonia in pigs [26]. In several bacterial species, it has been established that the amount of capsular polysaccharide is a major factor in virulence [92] and it decreases significantly with in vitro passages [93]. Furthermore, despite increasing evidence supporting the existence of a polysaccharide capsule in several *Mycoplasma* species, it is not yet known if the material is synthesized by the bacteria or imported from the medium for most species [94]. Recent reports have even shown the capacity of species from the mycoides cluster to assemble a capsule from exogenous phosphorylated glucose [79, 95]. However, no homolog proteins related to these activities were found in none of the species studied in this work. For the reasons discussed above, we were not able to introduce the capsule production itself in the models; however, we tried to relate its possible existence with the substrates that might be used to produce it. The composition of capsular polysaccharides has been related to the level of pathogenicity in other bacteria. For instance, highly virulent *Escherichia coli* strains seem to have practically nonimmunogenic capsular material, due to the fact that these antigens are similar or identical to the ones found in the host [96]. The structure of the K5 antigen from *E. coli* capsules is basically GlcNAc and glucuronic acid (GlcA) in a molar ratio of 1:1 [97]. Erlinger and collaborators [98] identified heparan sulfate (HS) as the predominant glycosaminoglycan in the porcine respiratory tract and the most common disaccharide unit within HS is GlcNAc linked to GlcA. Since *M. hyopneumoniae* and *M. flocculare* might be able to import GlcNAc unchanged, they may use it for the composition of its capsule; *M. hyorhinis*, on the other hand, imports sialic acid, and might use it directly to produce its own. Of course this is highly speculative, and we are not sure if all these hypotheses indeed happen in vivo.

The metabolism of glycerol is the final major difference between the pathogenic *M. hyopneumoniae* and *M. hyorhinis* and the non-pathogenic *M. flocculare*. Such metabolism and the production of hydrogen peroxide by the action of GlpO are essential for the cytotoxicity of *M. pneumoniae* [35] and *M. mycoides* subsp. *mycoides* [36]. Particularly, the cytotoxicity of *M. mycoides* subsp. *mycoides* is due to the translocation of the hydrogen peroxide into the host cells. This is only possible because of the close proximity to the host cells along with the membrane-bound enzyme. Highly conserved homolog genes to this enzyme were only found in the genomes of the pathogenic species studied here. Whether or not these enzymes from *M. hyopneumoniae* and *M. hyorhinis* are indeed capable of the same activities as GlpO is not yet confirmed; however, the high similarity may be an indication that this trait is essential for the pathogenicity of these two species. This may also explain why both *M. hyopneumoniae* and *M. flocculare* can adhere to the cilia of tracheal epithelial cells, but only the adhesion of *M. hyopneumoniae* results in tissue damage [33]. Moreover, while *M. hyorhinis* has in the reconstructed models three ways of uptaking glycerol, *M. hyopneumoniae* seems to have five, and this may reflect in its enhanced pathogenicity.

## Conclusions

We presented in this work an overview of the differential metabolism of *M. hyopneumoniae*, *M. hyorhinis* and *M. flocculare* using different approaches. The reconstructed models showed some distinctions among the species, namely the myo-inositol metabolism for *M. hyopneumoniae*, the wider uptake of carbohydrates for *M. hyorhinis* and the usage of glycerol as a carbon source for the two pathogenic species. The models also served as a basis for all the assumptions made for the experimental data. Metabolic profiling of both complex and defined media pointed to new differences that we were not able to identify based solely on the sequenced genomes. The major ones were related to the pyruvate conversion to acetate, which appeared to be higher in *M. hyopneumoniae* and *M. flocculare* than in *M. hyorhinis*. After we performed growth tests using defined media that hypothetically would lead to bacterial growth *in silico*, we assessed that some cofactors or metabolites were probably being delivered in the wrong form to the mycoplasmas. The environmental context may explain the differences between the *in silico* models and the in vivo behavior.

Whether the main differences among the species we reported here (summarized in Additional file 1: Table S8) are related to virulence or pathogenicity have not yet been addressed experimentally, but it is tempting to speculate. The same factors that may enhance virulence of *M. hyopneumoniae* may help the commensal species *M. flocculare* to better survive inside the host.

All these *in silico* and in vivo metabolic differences among *M. hyopneumoniae*, *M. hyorhinis* and *M. flocculare* might influence the different levels of pathogenicity in each of them. However, it is highly likely that gene regulation may directly interfere in the metabolism and pathogenicity and may be related to many other aspects still unaccounted for. One of our future goals is therefore to understand and integrate gene regulation into the metabolic models. Upcoming experiments will aim at testing the hypotheses formulated here, particularly those related to the metabolisms of glycerol and myo-inositol. We also intend to better understand the habitat of these species, and the possible metabolic and genetic dialogues with the host and other bacteria present in this environment. Either way, this work serves as a basis for the study of the differential metabolism and pathologies caused by the swine lung mycoplasmas and may help to propose ways to prevent disease development in the future.

## Availability of data and material

All models from this work are available in Additional file 5 and online at the MetExplore database (http://metexplore.toulouse.inra.fr/metexplore2/).

## Additional files

Additional file 1 Supplementary Tables. **Table S1.** Defined media composition used for the cultivation of *M. hyopneumoniae*, *M. hyorhinis* and *M. flocculare*. **Table S2.** Complete list of reactions and metabolites from all models. **Table S3.** List of all metabolites included in the final biomass composition. **Table S4.** Distribution of GPR associations and exclusive GPR associations by metabolic pathways. **Table S5.** Proposition of correct precursors and cofactors for a new defined medium. **Table S6.** NMR metabolomics: Metabolites detected in the complex Friis and Yus+ media for the three swine mycoplasmas. **Table S7.** Prediction of essential reactions in the pan-network. **Table S8.** Differential metabolism overview. (XLSX 297 kb)

Additional file 2 Biomass reaction assembly from reconstructed models. Pdf file containing all steps necessary to the assembly of the biomass reaction for all species. (PDF 906 kb)

Additional file 3 Abbreviation of metabolites and EC numbers from Figs. 3, 4 and 7. Spreadsheet containing the lists of all abbreviations and EC numbers used in Figs. 3 and 4 separately. (XLSX 22kb)

Additional file 4 Prediction of transporters. Pdf file containing all assumptions for the *in silico* prediction of transporters. (PDF 789 kb)

Additional file 5 Models in SBML format. Compressed file (.zip) containing all SBML models from this work (.sbml). (ZIP 407 kb)

## Abbreviations

ATP-binding cassette; ACP: Acyl-carrier protein
BLASTp: blast local alignment search tool (protein)
DEM: dead-end metabolite
CCU: color changing unit
CPMG: Carr-Purcell-Meiboom-Gill
EC number: enzyme-code number
FBA: flux balance analysis
FID: free induction decay
GAM: growth associated maintenance
GO: gene ontology
GPR: gene-protein-reaction association
HCA: hierarchical clustering analysis
HSQC spectroscopy: heteronuclear single-quantum correlation spectroscopy
MIF: major facilitator protein
MW: molecular weight
NGAM: non-growth associated maintenace
NMR: nuclear magnetic resonance
NOESY: nuclear overhauser effect spectroscopy
PCA: principal component analysis
Pfam database: protein-families database
PTS: phospho-transferase transport system
SBML: systems biology markup language
TMSP: trimethilsilylpropionate
TOCSY: Total-quantum correlation spectroscopy
TU: transcriptional unit.
Enzyme abbreviations: ABC-Gly: ABC-glycerol transport system
GlyF: glycerol facilitator protein
GlpO: glycerol-3-phosphate
GlpU: glycerophosphodiester transporter
Fba: fructose-biphosphate-aldolase
IolJ: 5-Dehydro-2-deoxy-phosphogluconate aldolase
Ldh: lactate dehydrogenase
Mdh: malate dehydrogenase
Pdh A/B/C/D: pyruvate dehydrogenase complex subunit A/B/C/D
UlaG: L-Ascorbate-6-phosphate lactonase
Metabolite abbreviations used in the text (the complete list of metabolites may be found in Additional file 4): 2KG: 2-Oxoglutarate
AcCoA: Acetyl-coenzyme-A
CoA: coenzyme-A
DHA: dihydroxyacetone
DHAP: dihydroxyacetone-phosphate
F6P: fructose-6-phosphate
GlcNAc: N-acetyl-glucosamine
NAD/NADH: nicotinamide adenine dinucleotide
NADP/NADPH: nicotinamide adenine dinucleotide phosphate
MeTHF: 5,10-methylene-tetrahydrofolate
SucCoA: succinyl-coenzyme-A
