# Supplementary material for: Insights on the virulence of swine respiratory tract mycoplasmas through genome-scale metabolic modeling
Source: BMC Genomics. 2016 May 13;17:353. doi: 10.1186/s12864-016-2644-z (PMC4866288; doi:10.1186/s12864-016-2644-z)
Supplement: Additional file 2 — Biomass reaction assembly from reconstructed models. Pdf file containing all steps necessary to the assembly of the biomass reaction for all species. (PDF 906 kb) [file 12864_2016_2644_MOESM2_ESM.pdf]

Biomass Composition and Biomass Reaction Assembly

Biomass Composition

The biomass reaction drained all precursors (in their molar biological ratios) into biomass. Based on the mentioned macromolecule composition (Material and Methods Section), we were able to separately calculate the subsystems of biomass components per gram of biomass dry weight (g DW). Nucleotide and protein composition were derived from genome sequence; lipid, carbohydrate, ions and cofactors were based on literature data.

Table ST1: Average cellular composition of *M. flocculare*, *M. hyorhinis* and *M. hyopneumoniae*.

	% (w/w)	% (g/g DW)
**DNA**	6,00	0,0600
**RNA**	12,00	0,1200
**Proteins**	55,00	0,5500
**Lipids**	15,00	0,1500
**Cofactors**	1,68	0,0168
**Ions**	3,44	0,0344
**Carbohydrates**	6,88	0,0688

DNA composition

An average DNA composition based on the genome sequence for all strains was defined (Table ST2).

Table ST2: GC and AT content from all strains (except for MHR17981). Since there was no genome announcement for MHR17981 and no complete chromosome, this strain was not taken into account.

	%AT	%CG
MHP168	71,54	28,46
MHP168L	71,54	28,46
MHP232	71,51	28,49
MHP7422	71,50	28,5
MHP7448	71,50	28,5
MHPJ	72,11	27,89
MHRHUB1	74,12	25,88
MHRGDL1	71,91	28,09
MHRSK76	72,21	27,79
MRH17981	NA	NA
MFL27716	71,10	28,90
MFL27399	71,04	28,96
**Average**	**71,83**	**28,17**

The average molecular weight of a nucleotide residue (inside DNA) was calculated from the molecular weight of nucleotides (Table ST3).

Table ST3: Average DNA content from all strains and average molecular weight of nucleotide residue in DNA.

	% (mol/mol)	MW of residue (g/mol)
**dAMP**	35,915	311,19
**dTMP**	35,915	302,18
**dGMP**	14,085	327,19
**dCMP**	14,085	287,17
**Average MW of DNA residue**		**306,82**

Based on an average molecular weight of 306.82 g/mol of DNA residue, we concluded that 0.06g of DNA corresponded to 0.1955 mmol of DNA per gdW. The concentration of each base in mmol/gdW was estimated and may be found in Table 3.

RNA composition

The RNA composition was calculated similarly to DNA. The GC and AT content was based only on the open reading frames (ORFs) annotated in genbank files (Table ST4). An average nucleotide molecular weight inside the RNA molecules was calculated and can be found in Table ST5.

Table ST4: Theoretical RNA nucleotide content from all strains.

	AMP	UMP	CMP	GMP
MHP168	38,47	32,98	13,73	14,82
MHP168L	38,43	32,97	13,77	14,83
MHP232	38,37	32,94	13,87	14,82
MHP7422	38,37	32,99	13,80	14,84
MHP7448	38,30	33,01	13,85	14,84
MHPJ	38,40	32,88	13,88	14,84
MHRHUB1	40,14	33,16	12,85	13,85
MHRGDL1	39,86	33,42	12,86	13,86
MHRSK76	40,25	33,12	12,84	13,79
MRH17981	40,65	33,07	12,56	13,72
MFL27716	38,06	32,09	14,45	15,40
MFL27399	37,96	32,18	14,46	15,40
**Average**	**38,94**	**32,90**	**13,58**	**14,58**

Table ST5: Average RNA content from all strains and average molecular weight of the nucleotide residue in RNA.

	% (mol/mol)	MW of residue (g/mol)
**AMP**	38,94	327,19
**UMP**	32,9	304,15
**GMP**	14,58	343,19
**CMP**	13,58	303,17
**Average MW of RNA residue**		**318,68**

Based on an average molecular weight of 318.68 g/mol of RNA residue, we concluded that 0.12g of RNA, corresponded to 0.3765 mmol of RNA per gdW; the concentration of each base in mmol/gdW was estimated and is found in Table 3.

Amino acid composition

Amino acid composition accounted for all cellular proteins and was estimated by sequence analysis of translated mRNA (Table ST6). The average molecular weight of an amino acid residue was calculated and can be found in (Table ST7).

ST6: Amino acid content from all analyzed strains.

	MHP168	MHP168L	MHP232	MHP7422	MHP7448	MHPJ	MHRHUB1	MHRGDL1	MHRSK76	MHR17981	MFL27716	MFL27399	Avg
Alanine	5,12	5,07	5,05	5,05	4,97	5,04	5,06	5,05	5,05	5,06	5,34	5,35	**5,10**
Arginine	2,96	2,97	2,97	2,97	3,00	2,97	2,92	2,94	2,89	2,87	2,89	2,91	**2,94**
Asparagine	7,66	7,64	7,62	7,62	7,62	7,61	7,61	7,60	7,62	7,61	7,71	7,68	**7,63**
Aspartic Acid	4,74	4,77	4,78	4,78	4,85	4,80	4,87	4,83	4,89	4,90	4,62	4,67	**4,79**
Cysteine	0,43	0,42	0,41	0,41	0,41	0,41	0,41	0,42	0,41	0,41	0,45	0,42	**0.42**
Glutamic Acid	6,44	6,47	6,47	6,47	6,52	6,48	6,49	6,48	6,50	6,50	6,36	6,44	**6,47**
Glutamine	3,99	4,01	4,04	4,05	4,06	4,06	4,12	4,08	4,14	4,14	3,92	3,94	**4,05**
Glycine	4,57	4,57	4,58	4,58	4,62	4,59	4,50	4,54	4,47	4,43	4,58	4,57	**4,55**
Histidine	1,24	1,23	1,22	1,22	1,22	1,22	1,27	1,25	1,29	1,31	1,27	1,28	**1,25**
Isoleucine	9,91	9,91	9,90	9,88	9,86	9,87	9,71	9,80	9,66	9,63	9,88	9,89	**9.83**
Leucine	9,93	9,96	9,97	9,97	9,95	9,95	9,92	9,94	9,90	9,90	9,84	9,81	**9.92**
Lysine	10,75	10,77	10,77	10,76	10,70	10,76	10,74	10,73	10,74	10,73	10,68	10,74	**10,74**
Methionine	1,25	1,25	1,25	1,25	1,29	1,25	1,28	1,27	1,29	1,30	1,24	1,23	**1,26**
Phenylalanine	6,93	6,93	6,93	6,92	6,89	6,91	6,76	6,83	6,71	6,68	6,93	6,88	**6,86**
Proline	3,00	3,01	3,03	3,03	3,07	3,04	2,94	2,99	2,90	2,88	2,98	2,96	**2.99**
Serine	7,11	7,09	7,09	7,09	7,04	7,09	7,11	7,09	7,12	7,12	7,18	7,10	**7,10**
Threonine	4,54	4,52	4,51	4,52	4,44	4,51	4,67	4,60	4,74	4,78	4,66	4,66	**4,60**
Tryptophan	0,99	0,99	1,00	1,00	1,03	1,00	1,00	1,00	1,00	1,00	0,99	0,95	**0,99**
Tyrosine	3,67	3,68	3,69	3,69	3,75	3,70	3,72	3,71	3,74	3,75	3,61	3,64	**3,70**
Valine	4,76	4,74	4,73	4,74	4,71	4,73	4,90	4,83	4,95	5,01	4,87	4,87	**4.82**

Table ST7: Average amino acid content from all strains and average molecular weight of amino acid residue.

	% (mol/mol)	MW of residue (g/mol)
**Alanine**	5,10	71,08
**Arginine**	2,94	157,19
**Asparagine**	7,63	114,10
**Aspartic Acid**	4,79	114,08
**Cysteine**	0,42	103,14
**Glutamic Acid**	6,47	128,11
**Glutamine**	4,05	128,13
**Glycine**	4,55	57,05
**Histidine**	1,25	137,14
**Isoleucine**	9,83	113,16
**Leucine**	9,92	113,16
**Lysine**	10,74	129,18
**Methionine**	1,26	131,20
**Phenylalanine**	6,86	147,17
**Proline**	2,99	97,12
**Serine**	7,10	87,08
**Threonine**	4,60	101,10
**Tryptophan**	0,99	186,21
**Tyrosine**	3,70	163,17
**Valine**	4,82	99,13
**Average MW of Amino Acid Residue**		**114,99**

Based on an average molecular weight of 114.99 g/mol of amino acid residue, we concluded that 0.55g of proteins corresponded to 4.78 mmol of proteins per gdW; the concentration of each amino acid in mmol/gdW was estimated and may be found in Table 3.

Lipid composition

Lipids were divided in three categories: sterols, phospholipids and glycolipids [maniloff1992,Kornspam2012]. Phospholipids and glycolipids were separated into two fractions: elementary portion and fatty acid radicals (Figure ST1). Based on the fatty acid composition [Chen1992,maniloff1992,Kornspam2012], we were able to estimate an average fatty acid molecular weight to incorporate into the elementary lipids portion. The fatty acids associated with elementary portions of lipids seem to mimic the total fatty acid composition of the organism and are dependent on the composition of the culture medium [Dahl1980, maniloff1992].

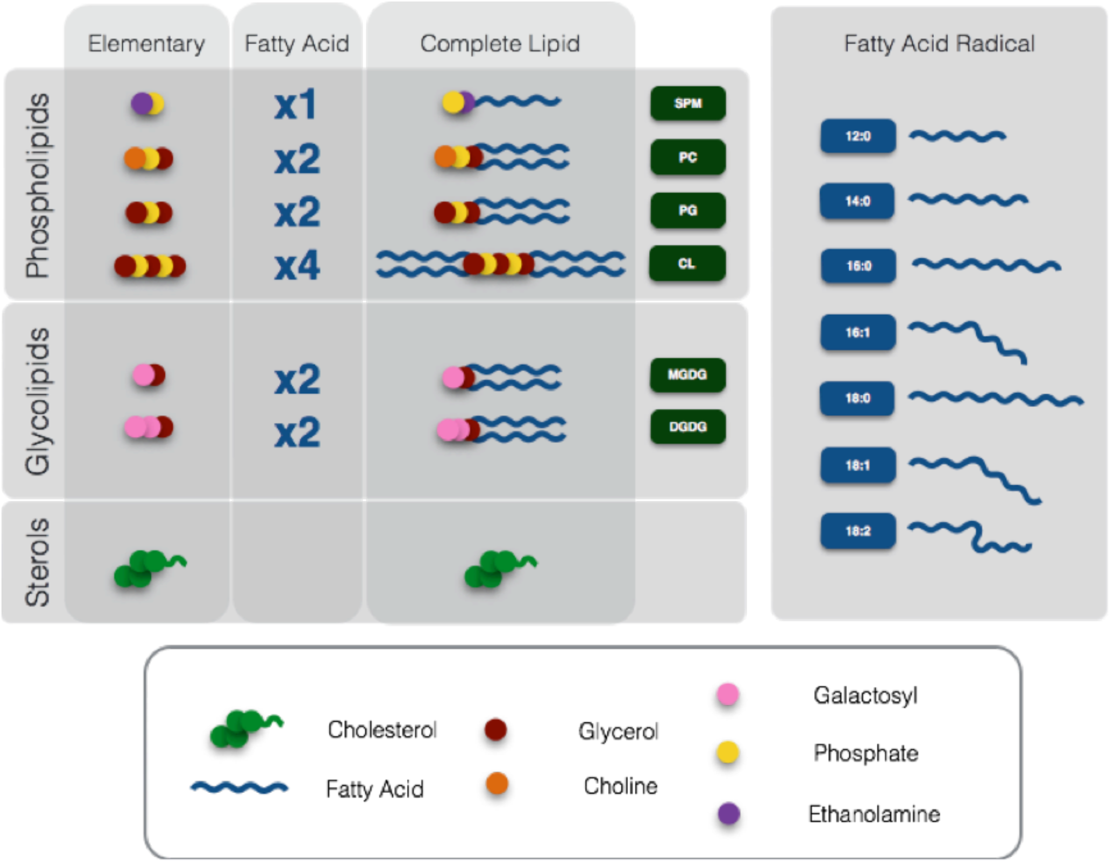

Figure ST1: Lipids were separated into elementary portion and fatty acid radicals. Phospholipids and glycolipids were taken into account as an elementary part and two or more fatty acids: Sphingomyelin (SPM), phosphatidylcholine (PC), phosphatidylglycerol (PG), monogalactosyldiacylglycerol (MGDG) and digalactosyldiacylglycerol (DGDG) are composed by the elementary fractions and two fatty acid radicals, cardiolipin (CL) consists of its elementary portion and 4 fatty acid radicals. Cholesterol has no fatty acid radicals. Fatty acid radicals can occur in saturated (XX:1 or XX:2) or unsaturated form (XX:0). For nomenclature and details see Table ST10. Multiple combinations of fatty acids and elementary portions can occur, this is why we treated each of them separately.

The main difference between *M. hyorhinis* and *M. hyopneumoniae* is that *M. hyorhinis* does not possess glycolipids [maniloff1992] while both glycolypids, monogalactosyldiacylglycerol (MGDG) and digalactosyldiacylglycerol (DGDG) were previously detected in *M. hyopneumoniae* [Chen1992]. Since no information was available for *M. flocculare*, we extrapolated the data from *M. hyopneumoniae* and included both glycolipids in its biomass composition.

Table ST8: Fatty acid saturation, composition and average fatty acid molecular weight in *M. hyopneumoniae* (data extracted from [Chen1992] and [maniloff1992]).

Fatty Acids	Size:Saturation	% (w/w)	MW (g/mol)
Lauric	12:0	8,8	155,3
Myristic	14:0	7,6	195,4
Palmitic	16:0	32,9	211,4
Palmitoleic	16:1	1,7	209,4
Stearic	18:0	7,0	239,5
Oleic	18:1	34,5	237,4
Linoleic	18:2	7,5	235,4
Average MW of Fatty Acid residue			217,96

Table ST9: Elementary molecular weight for each lipid (excluding fatty acid radicals).

Lipid	MW (g/mol)
Cholesterol	370,65
CL	299,15
PC	506,20
PG	310,22
SPM	491,62
MGDG	308,24
DGDG	470,38

Table ST10: Overall lipid composition and average molecular weight of a complete lipid of *M. hyopneumoniae* (MHP), *M. hyorhinis* (MHR) and *M. flocculare* (MFL).

Lipid	MW (g/mol)	MHR	MHP/MFL
		% (w/w)	% (w/w)
Cholesterol	370,65	50,0	25,0
CL	1378,00	20,7	20,7
PC	746,11	6,3	6,3
PG	735,04	6,2	6,2
SPM	709,57	16,8	16,8
MGDG	744,13	0	16,7
DGDG	906,28	0	8,4
Average Complete Lipid MW (g/mol)		682,0	789,3

* MW includes the elementary molecular weight of each lipid (Table ST9) and the average fatty acid molecular weight in the correct quantities (as described in Figure ST1).

** When present, the percentage of phospholipids equals the combined percentages of neutral and glycolipids. When absent, neutral lipid content equals the phospholipid content [maniloff1992].

*** Lipid composition was based on [Kornspan2012], [maniloff1992] and [Chen1992]. We corrected the composition of M. hyopneumoniae and M. flocculare to accommodate the presence of glycolipids (MGDG and DGDG), but kept the relative ratio between phospholipids. CL: Cardiolipin; PC: Phosphatidylcholine; PG: Phosphatidylglycerol; SPM: Sphyngomyelin; MGDG: Monogalactosyldiacylglycerol; DGDG: Digalactosyldiacylglycerol.

Based on an average molecular weight of 682.0 g/mol for M. hyorhinis, we estimated that 0.15g of lipids corresponded to 0.220 mmol of lipids per g DW; the concentration of each elementary lipid and each fatty acid in mmol/g DW was estimated and may be found in Table 3. Based on an average molecular weight of 789.3 g/mol for *M. hyopneumoniae* and *M. flocculare*, we estimated that 0.15g of lipids corresponded to 0.19 mmol of lipids per g DW; the concentration of each elementary lipid and each fatty acid in mmol/g DW was estimated and may be found in Table 3.

Ions and Cofactors

We adapted the ions and cofactors composition from the metabolic network available for M. pneumoniae [Wodke2013]. The concentration of each compound can be found directly in Table 3.

Carbohydrate composition

The structural unit for carbohydrate/polysaccharide fraction was fixed only as glucose for simplicity reasons. However, the polysaccharide composition in these species was not yet determined. The concentration of glucose can be found directly in Table 3.

Biomass Reaction Assembly

In order to create the biomass, the cell must unwind and replicate DNA, transcribe and degrade RNA, translate and modify proteins, among others. The approximate costs for several maintenance functions associated with growth (GAM) were calculated and are presented in Table ST11.

Table ST11: Energy requirements for biomass assembly (adapted from [Neidhartd1987]).

Synthesis and Processing		umol ATP/umol macromolecule	umol ATP/ gDW
DNA	Unwinding helix	1,000	195,60
	Proofreading	0,360	70,40
	Negative supercoiling	0,100	19,60
	Discontinuous synthesis	0,006	1,20
	Methylation	0,001	0,20
RNA	Discarding segments	0,380	143,10
	Modification	0,020	7,50
Protein	mRNA Synthesis	0,200	956,60
	Activation and incorporation	2,000*	9565,70
	Proofreading	0,100	478,30
	Assembly and modification	0,006	28,70
TOTAL umol of ATP			11466,90

* To adjust the ATP requirements and accommodate tRNA charging directly into the models, activation and incorporation were discounted 2 ATPs per umol of protein.

We also have to take into consideration that for every polymerization step (and every ATP used), we have extra substrates and products. For instance, even if we consider that in the DNA, cytosine is in the form of dCMP, the

reaction in vivo uses a dCTP as substrate and produces a pyrophosphate (PPi), incorporating a molecule of dCMP.

n dNTPs + n ATP => DNA + n PPi
n NTP + n ATP => RNA + n PPi
n amino acids + n ATP => 1 protein + n ADP + n Pi + nH2O

From all these estimated quantities, we were able to assemble the biomass reaction into the following form:

Biomass Precursors + 11,53 ATP + 6,11 H2O + Charged tRNAs => 1 Biomass + 11,46 ADP + 11,45 Pi + 11,46 H+ + 0,57 PPi + Uncharged tRNAs

where the stoichiometric coefficients for all biomass precursors can be found in Table 3 (with the exception of ATP, Pi and Ppi, which were adapted for ATP maintenance functions).
