# Supplementary material for: Insights on the virulence of swine respiratory tract mycoplasmas through genome-scale metabolic modeling
Source: BMC Genomics. 2016 May 13;17:353. doi: 10.1186/s12864-016-2644-z (PMC4866288; doi:10.1186/s12864-016-2644-z)
Supplement: Additional file 4 — Prediction of transporters. Pdf file containing all assumptions for the in silico prediction of transporters. (PDF 789 kb) [file 12864_2016_2644_MOESM4_ESM.pdf]

Transporters

Before the genome sequencing era, it was anticipated that Mollicutes, because of their small genomes, reduced metabolism and complex nutritional demands, should have a relative larger fraction of membrane transporters than large genome bacteria [Razin2002]. However, such expectations were not confirmed after the sequencing of several genomes; the transporter numbers seemed to be average when compared to other bacteria [Fraser1995, Himmelreich1996,Paulsen1998,Pollack2002].
An extensive search for transporters throughout all species was performed and is described below.

Active transporters

Active transporters use a chemical, electrical or solar source to drive solute transport [Saier2000]. Although uncharged solutes, such as sugars and aminoacids may cross the membrane coupled with protons, the proton motor force (pmf) detected in *A. laidlawii* was proposed to arise from proton pumps primarily from ATP hydrolysis [Cirillo1993]. Mycoplasmas in general, lacking both electron transport and a functional TCA cycle, use ATP as primary energy source, and in turn, have a great number of ATP-dependent transport systems (ATP binding cassette or ABC transport systems). ABC transporters are generally involved in a variety of substrates such as amino acids, sugars, peptides and toxins. In fermentative species of *Mycoplasma*, sugar is thought to be transported through group translocation transport systems (PEP:Pyruvate phosphotransferase systems or PTS). Import through a PTS results in phosphorylation of carbohydrate substrate whereas ABC transport does not modify the substrate (Fig ST1), which means that ABC transport of carbohydrates requires more energy than PTS transport [Buckwalter2012].

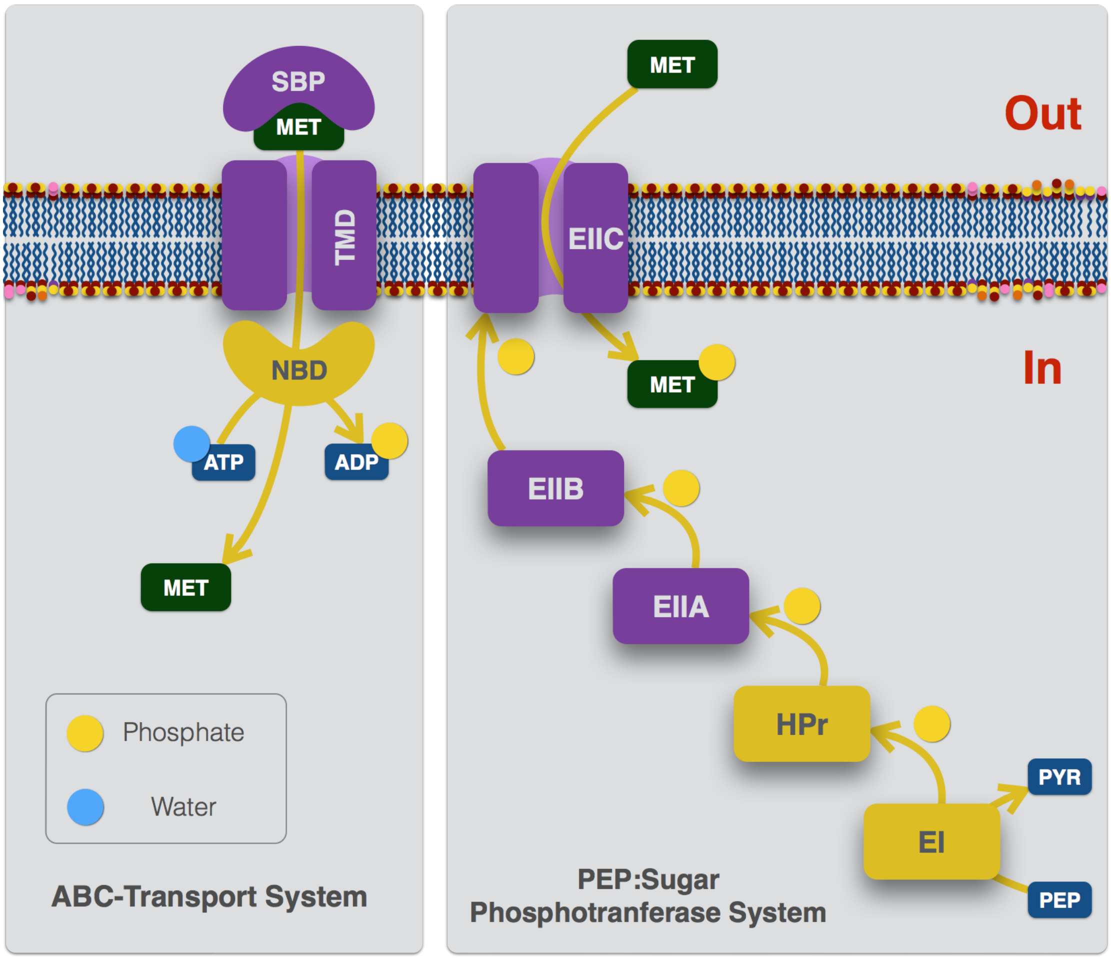

Figure ST1: Schematic representation of an ABC transport system and a PTS. Components that are common to other transport systems are depicted in yellow and specific-substrate proteins are depicted in purple. Substrate (MET) is depicted in green and cofactors in blue (ATP, ADP, PEP, PYR). (a) ABC import systems are composed of 3 separate portions, a substrate binding protein (SBP), two transmembrane domains (TMD, usually heterodimers) and two nucleotide binding domains (NBD, usually homodimers). (b) PTS is composed by two general proteins, phophocarrier (HPr) and enzyme I (EI), common to all systems; Enzymes IIA (EIIA), IIB (EIIB) are cytoplasmic phosphocarriers and enzyme IIC (EIIC) forms a membrane channel.

ABC transport systems:

ABC transporters are integral membrane proteins that enable active transport across the lipid membrane against a concentration gradient of substrates, by using energy of ATP hydrolysis [Moussatova2008]. ABC transporters are composed of two transmembrane permeases (TMDs), two nucleotide binding domains (NBDs), which are cytoplasmic proteins able to hydrolize a nucleotide triphosphate and a substrate binding domain, usually a lipoprotein which is not necessary if the substrate is delivered by the lipid bilayer.

The results for the search of ABC transporters are summarized in table ST1, for one member of each species: *M. hyorhinis* strain HUB1 (MHRHUB1), *M. hyopneumoniae* strain 7448 and *M. flocculare* ATCC 27399. Complete systems had at least one TMD and one NBD that were either in the same gene or in separate genes. But, as proposed in other bacteria, several of the incomplete ABC systems may be functional, using components from the complete systems [Tchieu2001]. We also found a great number of lipoproteins with no assigned function in the neighborhood of some of these ABC cassettes. These conserved, but not characterized, proteins were proposed as possible substrate binding proteins whenever a SBP was missing from the cassette (Fig ST2).

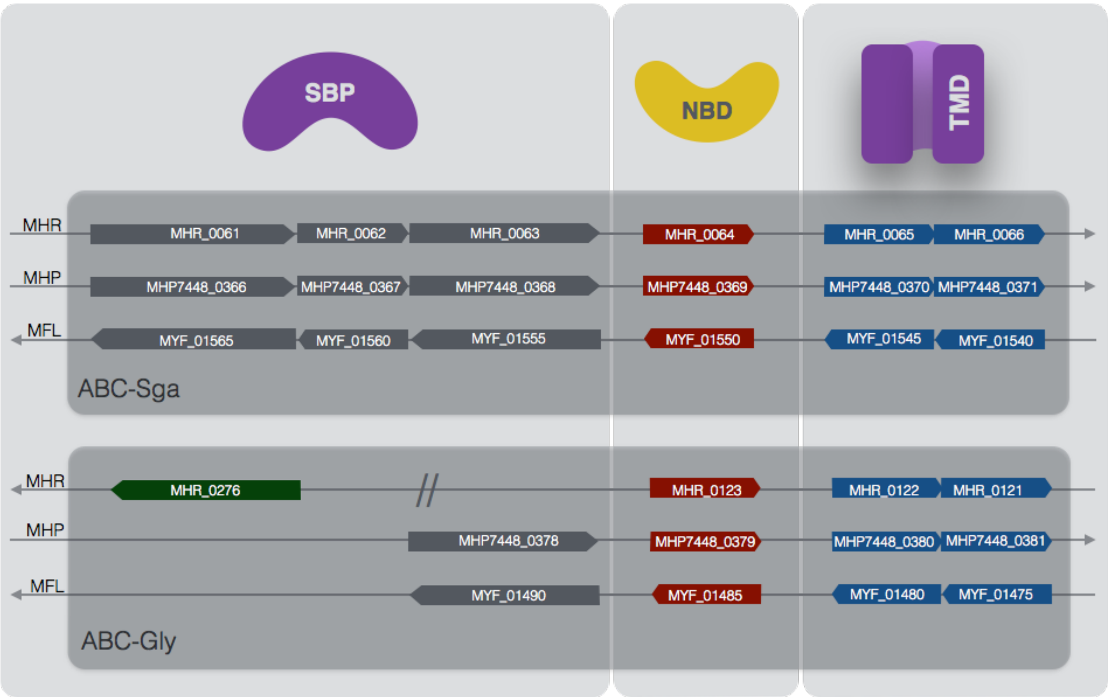

Figure ST2: Gene context and components of two ABC transport systems in *M. hyorhinis* (MHR), *M. hyopneumoniae* (MHP) and *M. flocculare* (MFL). Only one strain from each species was chosen (MHR strain HUB-1, MHP strain 7448, MFL ATCC 27399). The nonspecific sugar ABC transport system has three conserved lipoproteins upstream the ATP-binding protein, whereas the specific glycerol ABC transport system has only one substrate binding protein. In blue, annotated genes coding for transmembrane domain proteins; in red, annotated genes coding for nucleotide-binding proteins and in green, annotated genes coding for substrate binding protein. In deep grey, annotated genes coding for lipoproteins with unknown function; in this work these lipoproteins were proposed to act as substrate binding proteins, based on gene context.

Table ST1: ABC systems present in *M. hyorhinis*, *M. flocculare* and *M. hyopneumoniae*. Only three strains are presented here: MHRHUB1, MHP7448 and MFL27399.

ABC-Systems			ABC Components					
Substrate	Abbr	Organism	NBD		TMD		SBP	
Sugar	ABC-Sga	MHRHUB1	MHR_0064		MHR_0065	MHR_0066	MHR_0061	MHR_0062| MHR_0063
		MHP7448	MHP7448_0369		MHP7448_0370	MHP7448_0371	MHP7448_0366	MHP7448_0367| MHP7448_0368
		MFL27399	MYF_01550		MYF_01540	MYF_01545	MYF_01555	MYF_01560| MYF_01565
Fructose/ Arabinose/ Galactose/Xylose	ABC-Fru	MHRHUB1	MHR_0163		MHR_0164		MHR_0162	
		MHP7448	MHP7448_0514		MHP7448_0515		MHP7448_0513	
		MFL27399	MYF_00865		MYF_00860		MYF_00870	
Maltose/ Maltodextrin	ABC-Mal	MHRHUB1	MHR_0194		MHR_0195	MHR_0196	MHR_0487	
		MHP7448						
		MFL27399						
Myo-inositol	ABC-Myo	MHRHUB1						
		MHP7448	MHP7448_0231		MHP7448_0233		MHP7448_0234	
		MFL27399						
Glycerol	ABC-Gly	MHRHUB1	MHR_0123		MHR_0121	MHR_0122	MHR_0276	
		MHP7448	MHP7448_0379		MHP7448_0380	MHP7448_0381	MHP7448_0378	
		MFL27399	MYF_01485		MYF_01475	MYF_01480	MYF_01490	
Peptides	ABC-Pep	MHRHUB1	MHR_0319		MHR_0318		MHR_0318	
		MHP7448	MHP7448_0452		MHP7448_0453		MHP7448_0453	
		MFL27399	MYF_01280		MYF_01275		MYF_01275	
Oligopeptides 1	ABC-Opp1	MHRHUB1	MHR_0359	MHR_0360	MHR_0361	MHR_0362	MHR_0357	
		MHP7448	MHP7448_0215	MHP7448_0214	MHP7448_0213	MHP7448_0212	MHP7448_0217	
		MFL27399	MYF_02620	MYF_02615	MYF_02610	MYF_02605	MYF_02595	MYF_02630
Oligopeptides 2	ABC-Opp2	MHRHUB1	MHR_0635	MHR_0636	MHR_0637	MHR_0638	MHR_0639	
		MHP7448	MHP7448_0501	MHP7448_0502	MHP7448_0503	MHP7448_0504	MHP7448_0505	
		MFL27399	MYF_00935	MYF_00930	MYF_00925	MYF_00920	MYF_00915	
Nucleosides	ABC-Nucl	MHRHUB1	MHR_0073		MHR_0074	MHR_0075	MHR_0076	
		MHP7448	MHP7448_0605		MHP7448_0606	MHP7448_0607	MHP7448_0604	
		MFL27399	MYF_02910		MYF_02915	MYF_02920	MYF_02905	
Thiamine/ Phosphate/ Phosphonate	ABC-Pi	MHRHUB1	MHR_0624		MHR_0623		MHR_0625	
		MHP7448	MHP7448_0361		MHP7448_0362		MHP7448_0360	
		MFL27399	MYF_01410		MYF_01415		MYF_01405	
Cobalt	ABC-Co	MHRHUB1	MHR_0108	MHR_0109	MHR_0110			
		MHP7448	MHP7448_0263	MHP7448_0264	MHP7448_0265			
		MFL27399	MYF_01890	MYF_01895	MYF_01900			
Putrescine/ Sperimidine	ABC-Spd/ Put	MHRHUB1	MHR_0466		MHR_0467	MHR_0468		
		MHP7448	MHP7448_0540		MHP7448_0540	MHP7448_0541		
		MFL27399	MYF_00720		MYF_00715	MYF_00710	MYF_00725	
Multidrug/Mn/Zn	ABC-Mn/Zn	MHRHUB1	MHR_0020		MHR_0019			
		MHP7448	MHP7448_0469		MHP7448_0470			
		MFL27399	MYF_01155		MYF_01150		MYF_01160	MYF_01165
Toxin Secretion	ABC-Detox1	MHRHUB1	MHR_0310		MHR_0310			
		MHP7448	MHP7448_0160		MHP7448_0160			
		MFL27399	MYF_02330		MYF_02330			
Multidrug Efflux	ABC-Detox2	MHRHUB1	MHP7448_0664	MHP7448_0665	MHP7448_0664	MHP7448_0665		
		MHP7448	MHR_0149	MHR_0150	MHR_0149	MHR_0150		
		MFL27399	MYF_03280	MYF_03285	MYF_03280	MYF_03285		

* In yellow: Genes that had not been annotated as part of an ABC transport system;
* In blue: Gene was already associated to ABC transport system, but a new activity was proposed;

We were able to identify in all species complete putative ABC transporters for multidrug/toxin efflux (ABC-Detox1 and ABC-Detox2) and for import of sugars (ABC-Sga, ABC-Fru), oligopeptides (ABC-Opp1, ABC-Opp2), peptides (ABC-Pep), spermidine/putrescine (ABC-Spd/Put), phosphonate/phosphate/thiamine (ABC-Pi), cobalt (ABC-Co),

manganese/zinc (ABC-Mn/Zn) and glycerol (ABC-Gly). In the search of a possible nucleotide transporter we came across with a work from Nakhyung [Nakhyung2009], in which a system previously annotated as sugar ABC transport was experimentally validated as involved in nucleoside uptake in *M. bovis*. We found similar genes in the genomes of all species (ABC-Nucl). *M. hyopneumoniae* has an extra ABC transporter proposed for myo-inositol (ABC-Myo) and *M. hyorhinis* has a unique ABC transport for maltose/maltodextrins (ABC-Mal).

PTS:

Phosphotransferase systems are widely spread among bacteria. It consists of two cytoplasmic energy-coupling proteins (enzyme I and phosphocarrier, Fig X) and a range of substrate-specific enzymes II, which catalyze simultaneously phosphorylation and traslocation into the cell. The phosphorylation status of each component is also an indicator of carbohydrate availability and can be used as signals to environmental conditions [Kotrba2001].

A gene coding for enzyme I (EI) and for the phosphocarrier (HPr) were found in all strains from *M. hyorhinis*, *M. hyopneumoniae* and *M. flocculare*. Although the gene was not annotated in *M. hyorhinis* strain HUB-1, the genomic sequence is present and there is no other gene overlapping the region (table ST2).

Enzymes II (IIA, IIB and IIC) are specific for one or a few given substrates and although most of them are encoded as separate genes, several EII fusions exist [Kotrba2001] (one example is the PTS-Fru in all species studied here, table X). A summarized list of all PTS found in one member of each species is found in table ST3.

Table ST2: Genes coding for PTS general proteins phosphocarrier (HPr) and Enzyme I (EI) in *M. hyorhinis*, *M. flocculare* and *M. hyopneumoniae*.

Organism	HPr	Enzyme I
**MHP168**	MHP168_619	MHP168_482
**MHP168L**	MHP168L_619	MHP168L_482
**MHP232**	mhp_628	mhp_470
**MHP7422**	MHL_2667	MHL_1734
**MHP7448**	MHP7448_0609	MHP7448_0472
**MHPJ**	MHJ_0611	MHJ_0469
**MHRHUB1**	NC_014448[746585..746857]	MHR_0477
**MHRGDL1**	MYM_0652	MYM_0503
**MHRSK76**	MOS_701	MOS_540
**MRH17981**	VBIMycHyo71610_0382	VBIMycHyo71610_0229
**MFL27716**	MFC_00378	MFC_01092
**MFL27399**	MYF_02935	MYF_01135

* The HPr gene was not annotated in the genome of *M. hyorhinis* strain HUB1 (MHRHUB1), but the genomic sequence is present and conserved (chromosome alignment Supp Fig X, alignment X).

Table ST3: Genes coding for PTS specific enzymes II in *M. hyorhinis*, *M. flocculare* and *M. hyopneumoniae*.

Systems			PTS Components		
**Substrate**	**Abbr**	**Organism**	**IIA**	**IIB**	**IIC**
**Sugar**	**PTS-Sga**	MHRHUB1	MHR_0457	MHR_0458	MHR_0459
		MHP7448	MHR_0194	MHR_0195	MHR_0487
		MFL27399	MYF_01515	MYF_01510	MYF_01505
**Fructose**	**PTS-Fru**	MHRHUB1	MHR_0231	MHR_0231	MHR_0231
		MHP7448	MHP7448_0492	MHP7448_0492	MHP7448_0492
		MFL27399	MYF_02570	MYF_02570	MYF_02570
**Mannitol**	**PTS-Mtl**	MHRHUB1	MHR_0169		
		MHP7448	MHP7448_0548	MHP7448_0550	MHP7448_0550
		MFL27399		MYF_02645	MYF_02645
**N-Acetylglucosamine**	**PTS-GlcNAc**	MHRHUB1			
		MHP7448	MHP7448_0574	MHP7448_0574	MHP7448_0574
		MFL27399		MYF_02765	MYF_02765
**Ascorbate**	**PTS-Asc**	MHRHUB1			
		MHP7448	MHP7448_0554	MHP7448_0553	MHP7448_0552
		MFL27399	MYF_02665	MYF_02660	MYF_02655
**Glucose**	**PTS-Glc**	MHRHUB1		MHR_0602	
		MHP7448		MHP7448_0591	
		MFL27399			

Three complete phospho-transferase transport systems (PTS) were common to all species: one non-specific for sugar (PTS-Sga), one with specificity for fructose (PTS-Fru) and another for mannitol (PTS-Mtl). *M. hyopneumoniae*

and *M. flocculare* also shared two extra complete systems: one for Ascorbate (PTS-Asc) and one for N-acetylglucosamine (PTS-GNAc). *M. hyorhinis* and *M. hyopneumoniae* seem to have an extra IIB component specific for glucose and along with the other components of PTS-Sga were proposed to form a complete glucose PTS (PTS-Glc).

Other transport systems with unknown mechanisms were found throughout the genomes and can be seen in table ST4.

Table ST4: Other genes coding for transport systems in *M. hyorhinis*, *M. hyopneumoniae* and *M. flocculare*.

Transport Systems			Transport Components		
**Substrate**	**Abbr**	**Organism**	**Gene 1**	**Gene 2**	**Gene 3**
Glycerol	Gly-F	MHRHUB1			
		MHP7448	MHP7448_0358		
		MFL27399	MYF_01395		
Glycerophosphodiesters	glpU	MHRHUB1	pseudo MHR_0261		
		MHP7448	MHP7448_0302		
		MFL27399			
Nonspecific	MIF	MHRHUB1	MHR_0432		
		MHP7448	MHP7448_0136	MHP7448_0302	
		MFL27399	MYF_02200		
Aminoacids	Aa-F	MHRHUB1	MHR_0146	MHR_0529	MHR_0586
		MHP7448	MHP7448_0081	MHP7448_0113	MHP7448_0357
		MFL27399		MYF_00625	MYF_00390
Cobalt/Magnesium	CorC	MHRHUB1	MHR_0236		
		MHP7448	MHP7448_0643		
		MFL27399	MYF_03085		
Sodium/Phosphate	Trans-Na/Pi	MHRHUB1	MHR_0250		
		MHP7448			
		MFL27399			
Cation ATPase	Trans-Cation	MHRHUB1	MHR_0257		
		MHP7448	MHP7448_0268		
		MFL27399	MYF_01915		
Chromate	Trans-Chr	MHRHUB1	MHR_0326	MHR_0327	
		MHP7448	MHP7448_0145	MHP7448_0146	
		MFL27399	MYF_02255	MYF_02260	
Magnesium	Trans-Mg	MHRHUB1	MHR_0433		
		MHP7448	MHP7448_0487		
		MFL27399	MYF_00995		
Sialic Acid	Trans-SA	MHRHUB1	MHR_0451	MHR_0579	
		MHP7448			
		MFL27399			
Zync	Trans-Zn	MHRHUB1	MHR_0598		
		MHP7448	MHP7448_0090		
		MFL27399	MYF_00480		
Potassium	Trans-K	MHRHUB1	MHR_0650	MHR_0649	
		MHP7448	MHP7448_0546	MHP7448_0545	
		MFL27399	MYF_00685	MYF_00690	

The protein glpU, coded by gene MPN241 in *M. pneumoniae*, was recently described to act on the uptake of glycerophosphodiesters [Grosshennig2013]. We found homologs to this gene in all strains of *M. hyopneumoniae* and *M. hyorhinis* adjacent to the gene responsible for the metabolization of these substrates (GlpQ, Table ST5) [pneumoniaeGlpQ]. In *M. hyorhinis* strain HUB-1, the gene was annotated as a pseudo gene.

Table ST5: Genes from the metabolism of glycerophosphodiester are adjacent in genomes of all species analyzed of *M. hyopneumoniae* and *M. hyorhinis*.

	T059 and T102	R011 and R182
	**GplU**	**EC 3.1.4.2 (GlpQ)**
**MHP168**	MHP168_332	MHP168_331
**MHP168L**	MHP168L_332	MHP168L_331
**MHP232**	mhp316	mhp317
**MHP7422**	MHL_2654	MHL_1164
**MHP7448**	MHP7448_0302	MHP7448_0303
**MHPJ**	MHPJ_0294	MHJ_0295
**MHRHUB1**	pseudo MHR_0261	MHR_0262
**MHRGDL1**	MYM_0324	MYM_0323
**MHRSK76**	MOS_292	MOS_293
**MRH17981**	VBIMycHyo71610_0468	VBIMycHyo71610_0469

All species also possess several unspecific amino acid permeases (Aa-F), a glycerol facilitator protein (Gly-F, which is less efficient than ABC-Gly [glpDpneumoniae]) and a major facilitator protein, with unknown specificity (MIF). We could also find common transporters for cobalt and magnesium (CorC), chromate (Trans-Chr), magnesium (Trans-Mg), potassium (Trans-K), zinc (Trans-Zn) and a cation ATPase (Trans-Cation). *M. hyorhinis* had two extra transport systems: one for Sialic Acid (also known as N-Acetyl Neuraminate, Trans-SA) and one sodium phosphate cotransporter (Trans-Na/Pi).

Molecules that diffuse freely across membranes are small and nonpolar. Examples include carbon dioxide, molecular oxygen and nitrogen. Small, polar and uncharged molecules (such as water, ammonia, hydrogen peroxide and urea) can move across the plasma membrane through hydrophilic apertures. [cooper2007cell]. Weak acids also cross the membrane in the uncharged form [Cirillo1993].

As a general rule, enzymes in reduced genomes seem to gain more functions than their homologs in large genomes [Kelkar2013]; and this may be the case for missing transporters in *M. hyorhinis*, *M. hyopneumoniae* and *M. flocculare*.
